# The compounding effect: how neighbourhood dynamics shape police deployment and use of force

**DOI:** 10.1186/s40163-025-00258-6

**Published:** 2025-10-02

**Authors:** Amal Ali, Jasmine Oware, Jonathan Jackson, Ben Bradford

**Affiliations:** 1 Department of Methodology, London School of Economics and Political Science, Connaught House, Aldwych, London, WC2A 2AE UK; 2 Department of Security and Crime Science, University College London, London, UK 35 Tavistock Square, WC1H 9EZ

**Keywords:** TASER, Calls for service, Mental health, Police use of force, Neighbourhood characteristics

## Abstract

**Background:**

Calls for service are a major driver of police activity, yet their role in shaping the neighbourhood distribution of police use of force remains under-explored. Understanding where and why force is used requires examining how these calls cluster spatially—and how police interpret and respond to them.

**Methods:**

Using administrative data from an English police force (2018–2021), we analyse how neighbourhood characteristics—including mental health prevalence, racial composition, socioeconomic deprivation, residential instability, and crime rates—predict patterns of police deployment and use of force. We link call-for-service records with force incident data to trace the process from (a) call initiation to (b) priority grading, (c) TASER-equipped officer deployment, and (d) eventual use of force.

**Results:**

Calls for service are concentrated in disadvantaged neighbourhoods with elevated mental health need. These areas are also more likely to experience police use of force (including TASER). Yet public demand is refracted through institutional filters—such as call grading and officer deployment decisions—that concentrate how and where force is ultimately applied.

**Conclusions:**

Police use of force does not result from isolated actions, but from a sequence of decisions that compound the existing spatial clustering of public calls for service. Structural disadvantage, mental health distress and operational decision-making interact to concentrate force in already over-burdened communities. Addressing disproportionate use of force requires reform not only of police practice, but also of the upstream social conditions that generate repeated crisis response.

**Supplementary Information:**

The online version contains supplementary material available at 10.1186/s40163-025-00258-6.

## Introduction

The use of force remains one of the most scrutinised aspects of contemporary policing. Each encounter—whether involving physical restraint, baton strikes or TASER deployment—raises critical questions about necessity, proportionality and legitimacy. In the United Kingdom (UK), research shows that Black individuals and other ethnic minority groups are disproportionately subjected to TASER use (Dymond et al. 2024). This disparity reflects deeper structural inequalities in how policing is distributed—inequalities that are especially pronounced in areas marked by deprivation and disadvantage, where racialised and economically marginalised communities face heightened exposure to coercive police practices (TASERD, 2023; Bradford, 2017; Vomfell & Stewart, 2021; Suss & Oliveira, 2023). Similar patterns are evident in the United States (US), where neighbourhood characteristics such as poverty, crime and racial composition strongly predict both police presence and use of force (Lawton, 2007; Lee et al., 2010; Nouri, 2021; Roberts, 2004; Terrill & Reisig, 2003).

Understanding when and where police use force requires attention to the broader geography of police activity—including how deployments are patterned across neighbourhoods and how officers interpret the situations they encounter. A growing body of research highlights mental health as a key factor: communities marked by elevated psychological distress tend to experience more frequent police presence, which in turn increases the likelihood of force being used. In the US, areas with high rates of psychiatric crisis and substance misuse often see intensified police activity (Jindal et al., 2022; McLeod et al., 2020). In the UK, Kyprianides and Bradford (2024) found that mental health prevalence predicted police presence even after controlling for crime and deprivation. These findings suggest that police are not merely responding to criminal incidents—they are increasingly drawn into managing broader social and health-related crises, with mental health functioning as a key attractor of routine deployment.

Yet, the growing recognition of the link between mental health and police presence has not been matched by a clear understanding of the mechanisms through which these patterns emerge. One crucial but under-explored factor is calls for service, arguably one of the primary drivers of police activity. Much of the existing literature focuses on officer-initiated encounters such as street stops, overlooking the fact that many police–public interactions are reactive, triggered by calls from members of the public. Since force can only be applied when officers are physically present, the volume and nature of these calls likely shape both where and how often force is used. Neighbourhoods that generate more calls, particularly those involving violence, domestic disputes or mental health crises, may experience more frequent deployments and greater opportunities for force to be applied. This raises a key but under-examined question: how do calls for service cluster spatially, and to what extent do these patterns help explain the geographic concentration of police use of force?

In this study, we examine how the neighbourhood distribution of calls for service relates to the spatial patterning of police use of force, with a particular focus on TASER deployment. Our approach draws on the framework proposed by Guarnera et al. (2024), who argue that disparities in the justice system can emerge not from isolated decisions, but from sequences of institutional actions that cumulatively reinforce disadvantage. While our analysis does not explicitly focus on bias, we do test a parallel dynamic in the geography of coercive policing. Specifically, we ask: (1) are calls for service disproportionately concentrated in structurally disadvantaged neighbourhoods with elevated mental health need?; (2) are these calls filtered through organisational processes—such as call grading and the deployment of TASER-equipped officers—that shape the intensity of the response?; and (3) is force ultimately applied in the very neighbourhoods where this institutional sequence begins?

We find that police use of force is not explained solely by crime rates or officer discretion, but by a cumulative process that begins the moment a call for service is made. This process includes decisions about call grading and whether to dispatch TASER-equipped officers, and is shaped by patterns of public demand, organisational priorities and neighbourhood-level conditions—particularly mental health distress. At each stage, structural disadvantage and institutional practice appear to reinforce one another, such that the final distribution of force reflects not just isolated encounters, but the layered interaction of demand, deployment and context. As such, our study offers a focused, empirical application of Guarnera et al.’s (2024) framework—it illustrates how structural conditions and institutional routines combine to produce spatial disparities in coercive policing.

The rest of the paper proceeds as follows. We begin by reviewing existing research on police use of force and calls for service, drawing on routine activity theory and the social ecology of crime and deprivation. We then describe our data, methods and empirical findings. We show how routine policing practices can explain unequal outcomes, not through overt discrimination, but through a compounding sequence in which neighbourhood disadvantage, mental health need, public demand and institutional response interact to predict the spatial distribution of force. We conclude by reflecting on the study’s limitations and outlining directions for future research.

## Police use of force

In 2022/23, police in England and Wales recorded 958,356 use of force tactics across 659,372 incidents (Home Office, 2023a, 2023b). “Restraint”, including handcuffing, accounted for 63% of all tactics, with handcuffing alone representing nearly half of all reported force actions. The second most common category, “unarmed skills”, made up 23% of tactics and included techniques such as escorting, distraction strikes, and pressure point locks. Our focus in this study on the weapons used by police, particularly Conducted Energy Devices (CEDs), which includes TASER, and during the same period, 34,687 instances of “less-lethal” weapon use were recorded, 97% of which involved a CED (33,351). By comparison, firearms accounted for fewer than 1% of all force tactics (5890).

CEDs warrant distinct empirical attention for four main reasons. First, public opinion research suggests that weapon use is viewed as less acceptable than unarmed force (Kyprianides et al., 2021). Second, CEDs, particularly TASER, are now by far the most commonly used police weapons in England and Wales. Third, TASER deployment raises specific concerns about racial and ethnic disparities. Fourth, the rarity of firearms use in British policing—there were just 11 discharges of a firearm in 2022/23—makes it difficult to conduct meaningful analysis in that domain.

The number of officers authorised to carry and use CEDs in England and Wales has risen steadily over the past two decades (Elliott-Davies & Glorney, 2024). This expansion has fuelled ongoing debate, largely due to pronounced racial disparities in CED use (HMICFRS, 2021; Home Office, 2022; Independent Office for Police Conduct, 2021). Data from the year ending 31 March 2023 show that individuals from Black ethnic groups were subject to police use of force at three times the rate of White individuals, while Black individuals were 4.6 times more likely to be involved in a TASER deployment (Home Office, 2023a, 2023b).

Despite these disparities, much existing research focuses on individual and organisational factors, often overlooking the broader ecological contexts in which force is used. Do officers deploy force—particularly TASERs—differently across neighbourhoods? If so, which neighbourhood characteristics explain those patterns? The answers to these questions remain unclear. An overlooked factor is spatial variation in calls for service and it is to this that we now turn.

## Calls for service

Calls for service are a key catalyst for police action (Langton et al., 2023).^1^ In 2012/13, for example, there were 19.2 million incidents recorded by police in England and Wales, the majority initiated by public calls (College of Policing, 2015). Over eight million of these were 999 emergencies, with 38% eliciting an immediate or priority response, 42% resolved by telephone, and the remainder scheduled for later attendance. As Ashby (2020: 1055) observes: “servicing the demand produced by calls for service consumes a large part of available police resources.”

Despite the volume of calls for service, few UK-based studies have examined the types of neighbourhoods that generate them or how they shape the spatial distribution of police activity. Calls for service, police presence and local characteristics such as crime and socioeconomic deprivation are likely to be strongly correlated. Yet, decisions made at the call-handling stage play a crucial role in shaping how calls translate into field operations. In the UK, call handlers determine grades of seriousness, add contextual notes and decide which calls are prioritised, in what order, and by which officers (Simpson, 2021).

The relationship between calls for service and police response emerges from a dynamic interplay of social, economic and cultural factors. These include individual and neighbourhood-level tendencies to contact the police (Sampson & Bartusch, 1998; Xie & Baumer, 2019), internal police practices and resource allocation (Davies & Bower, 2020; Lum et al., 2022) and broader structural pressures that shape the incidents police are asked to handle (Dau et al., 2023). This leads to a central question: how do these various factors predict neighbourhood-level patterning of calls for service and, in turn, the use of force?

## Gaps in the literature: neighbourhood structure, mental health and policing

Recent US-based research highlights the importance of ecological context in shaping police activity, particularly the use of force. Studies consistently find that geographical and socio-demographic factors—such as crime rates, deprivation, population density, and ethnic composition—predict both the frequency and nature of police interactions (Lawton, 2007; Lee et al., 2010; Nouri, 2021; Roberts, 2004; Terrill & Reisig, 2003). Miller et al. (2024: 3), for example, analysed police use of force during service calls across a range of incident types—including domestic disputes, disorder, mental health crises, alarm responses, and crime follow-ups—while controlling for neighbourhood characteristics such as concentrated deprivation, ethnic composition, violent crime, residential instability, and response time.

Despite this substantial body of work, key gaps and inconsistencies remain. The role of community-level factors in explaining the spatial clustering of police use of force is still contested, as is the extent to which incident type shapes officer behaviour (MacDonald et al., 2003). Much of the existing research either examines use of force in broad terms or focuses exclusively on severe incidents, often neglecting less-lethal tactics such as TASER deployment. Moreover, the literature remains predominantly US-centric, with limited attention to other jurisdictions—including the UK.

International research—though still largely US-based—also suggests that police activity is disproportionately concentrated in places and among populations affected by mental health conditions (Kyprianides & Bradford, 2025). Certain locations such as social service agencies, hospitals, homeless shelters, low-rent hotels, and alcohol retailers frequently serve as focal points for police presence, often involving individuals in mental distress (Hallet et al., 2021; Hodgkinson & Andresen, 2019; Koziarski, 2023; Tartaro et al., 2024; Vaughan et al., 2016). These sites may draw police attention for reasons beyond mental health alone, increasing the likelihood of encounters for everyone using them. At the same time, behaviours associated with mental illness could provoke police intervention regardless of location, leading to disproportionate contact with these groups (Hodgkinson & Andresen, 2019; Koziarski et al., 2022; Livingston, 2016). In the UK, the limited availability of alternative services has exacerbated this pattern, often casting the police—frequently described as the “service of last resort”—in a default crisis response role. This has prompted reform initiatives such as the Right Care, Right Person model (Home Office, 2023a, 2023b).

These behavioural, ecological and structural dynamics suggest that neighbourhoods with elevated mental health need may generate higher volumes of calls for service and, in turn, more frequent police deployments, both of which could increase the likelihood of force being used. Yet UK-specific empirical evidence remains sparse. One of the few national-level studies, by Kyprianides and Bradford (2024), found that local mental health prevalence at the LSOA level outside London was associated with stop and search activity. This relationship persisted even after controlling for ethnicity, age, deprivation, crime rates and service availability. Within London, by contrast, stop and search was more closely linked to crime rates and ethnic diversity. While this work underscores the relevance of area-level mental health in shaping frontline police practices, it did not examine calls for service as a potential upstream driver, nor did it extend to other forms of police contact such as deployment decisions or use of force.

We also test an interaction effect between mental health and deprivation. While we have some data on how deprivation and mental health each relate to police activity, to our knowledge no research has tested whether these factors interact (in a statistical sense) to predict police response. There are however intuitive—if necessarily speculative—reasons to consider such interactions. We are in exploratory territory, so we note that interaction effects could plausibly operate in either direction. On one hand, in more deprived neighbourhoods, visible signs of mental distress may be seen by residents—and anticipated by officers—as symptomatic of broader instability, prompting higher-risk grading and more precautionary deployments, including TASER-equipped units. On the other hand, in less deprived areas, the same behaviours may appear more anomalous or disruptive in an otherwise orderly setting, similarly triggering concern and escalated response. In both cases, police responses to mental health-related incidents may be influenced by officers’ explicit and implicit understandings of what local communities perceive as threatening or unacceptable, and therefore what the appropriate police response is.

## Theoretical framing

We examine the geographical distribution of police use of force in the UK through the lens of two influential criminological frameworks: routine activities theory (Cohen & Felson, 1979) and the social ecology of crime and deprivation (Shaw & McKay, 1942; Sampson & Wilson, 1995). We address how force becomes concentrated in specific neighbourhoods, and whether the spatial patterning of police activity—shaped significantly by calls for service—structures this distribution, including TASER deployment. The destinations to which officers are sent, the organisational processes determining their deployment, and the decisions made upon arrival, may all influence the likelihood that force is used in ways that accrue and compound across time and place.

Routine activities theory highlights how use of force is a spatially and temporally specific event, only occurring when officers and members of the public converge in time and space. Miller et al. (2024) offer one of the clearest empirical applications of routine activities theory to police use of force. Analysing administrative data from Cincinnati, they model the likelihood of force during service calls using situational, organisational and neighbourhood-level variables. Rather than treating force as spontaneous or exceptional, they locate it within the structured rhythms of routine police work, particularly call triage and dispatch. Their research demonstrates how organisational decisions,such as call grading, unit assignment and whether to dispatch weapon-carrying officers materially predict the probability of force. Their findings support the idea that structure, patterns of deployment, decision-making and neighbourhood exposure governs the conditions under which coercion occurs, beyond just individual intent.

The co-location of officers and members of the public is central to our study. For force to be used, both must be present in the same space at the same time. Importantly, social ecology perspectives explain why incidents generating police intervention are not evenly distributed. When officers are more frequently summoned to certain neighbourhoods, particularly those characterised by high levels of deprivation, crime and mental health need residents in these areas are likely to face disproportionate exposure to policing, including the potential for force. The causal pathways underlying these dynamics from the volume and nature of calls for service, through call grading and TASER-equipped officer deployment, to the final decision to use force will be complex and multifaceted. Officers are not simply dispatched and then mechanically apply force, nor are police organisations passive responders to demand: they make strategic decisions about resource allocation.

Although a full account of these institutional pathways exceeds our scope, we begin from the premise that force is rarely a discrete or isolated event. Rather, it represents the cumulative outcome of sequential operational decisions, shaped by agency priorities, dispatch protocols, resource constraints and unit-level cultures. Yet within this complexity emerge clear patterns, grounded in the empirical reality that police are more active in areas with greater levels of crime, deprivation and health or social need (Kyprianides & Bradford, 2024; Suss & Oliveira, 2023).

Our framework suggests that observed patterns of force at the neighbourhood level stem substantially from how police respond to public demand. Neighbourhoods with high levels of deprivation, crime and mental health challenges generate more calls for assistance. This initial concentration of demand is then filtered through decisions made during call handling, as dispatchers assess urgency and assign priority. These choices—whether explicit or tacit—may reinforce the concentration of police resources in already heavily policed areas, amplifying the clustering of force.

The type of personnel and equipment dispatched adds another crucial dimension to this dynamic. Not all UK officers carry TASERs, and even fewer are authorised to use firearms. The decision to send TASER-equipped officers may be influenced by perceptions of risk, potentially resulting in disproportionate deployments to particular neighbourhoods. This means some areas experience not only more frequent police presence but also greater exposure to coercive tools simply due to the nature of the response.

Ultimately, then, whether force is used depends on the interaction between officers and members of the public, encounters shaped by the full sequence of decisions leading up to them, from the initial call to the presence of TASER-equipped personnel. This integrated theoretical approach allows us to examine how use of force may reflect not only crime levels or officer discretion but also broader institutional mechanisms that systematically direct police presence and tactics toward particular neighbourhoods, with mental health challenges serving as a previously underexamined factor in this process.

## The current study

To investigate the neighbourhood-level predictors of police demand and use of force (Lum, 2011), we integrate police-reported use of force records with calls for service data, including response priority grades, using Lower Layer Super Output Area (LSOA) geo-codes. Our analysis focuses on data from a single English police force over a 4-year period (January 2018 to December 2021), allowing us to examine how key neighbourhood characteristics shape multiple stages of police deployment and force application. We assess how factors such as mental health prevalence, racial composition, concentrated deprivation, residential instability and crime rates predict: (a) the volume of public calls for police service, (b) the priority response grades assigned to these calls, (c) the likelihood of deploying TASER-equipped officers and (d) the frequency of police use of force.

Beyond conventional neighbourhood indicators—such as deprivation, crime rates, residential stability and demographic composition—we also incorporate the Small Area Mental Health Index (SAMHI) (Daras K, Barr B 2021). Unlike individual mental health measures, SAMHI captures area-level mental health as a broader social, economic and environmental phenomenon, reflecting how neighbourhood conditions shape collective well-being. By estimating the effects of structural factors such as crime and deprivation at each stage of the process, we can assess whether mental health prevalence plays an independent role in shaping police demand, response prioritisation and the application of force. We can also test whether SAMHI interacts with deprivation to predict key outcomes. This approach offers a more comprehensive account of how social and environmental conditions contribute to neighbourhood disparities in police interactions.

We have six research questions:

RQ1: What neighbourhood characteristics are associated with calls for police service? How do police service demands vary across LSOA?

RQ2: What neighbourhood characteristics are associated with levels of priority given to calls for police service?

RQ3a: What neighbourhood characteristics are associated with police resource allocation, specifically deploying TASER-equipped units?

RQ3b: Do mental health and deprivation interact to predict the dispatch of TASER-equipped units?

RQ4a: Within calls involving TASER-equipped officers, what neighbourhood characteristics are associated with incidents that involve the use of force and/or TASER use?

RQ4b: Do mental health and deprivation interact to predict incidents that involve the use of force and/or TASER use?

## Methodology

### Data

We constructed a dataset using multiple data sources, primarily derived from police administrative records from a single police force area in England.^2^ This force area, which is one of the largest in the country in terms of geographical coverage, includes both densely populated urban centres and sparsely populated rural regions with diverse policing demands. The first dataset consisted of calls for service spanning 1 January 2018 to 31 December 2021, comprising 1,411,462 observations. This dataset included contextual information such as the date and time the call was received, the initial incident grade and type, geographical location and resource allocation details.

For the same 4-year period, we also obtained mandatory self-reported use of force records (N = 20,094). These records capture detailed information, including the characteristics of the individuals involved, officer demographics, and the tactics employed. The calls for service dataset was matched with use of force records using a unique identifier provided by the police agency’s analyst. A successful match was achieved for 10,298 use of force records, representing 51.25% of use of force incidents initiated by a call for service. This matching rate highlights that approximately half of all use of force incidents arise from proactive or other policing activities rather than calls for service.

**Fig. 1:**
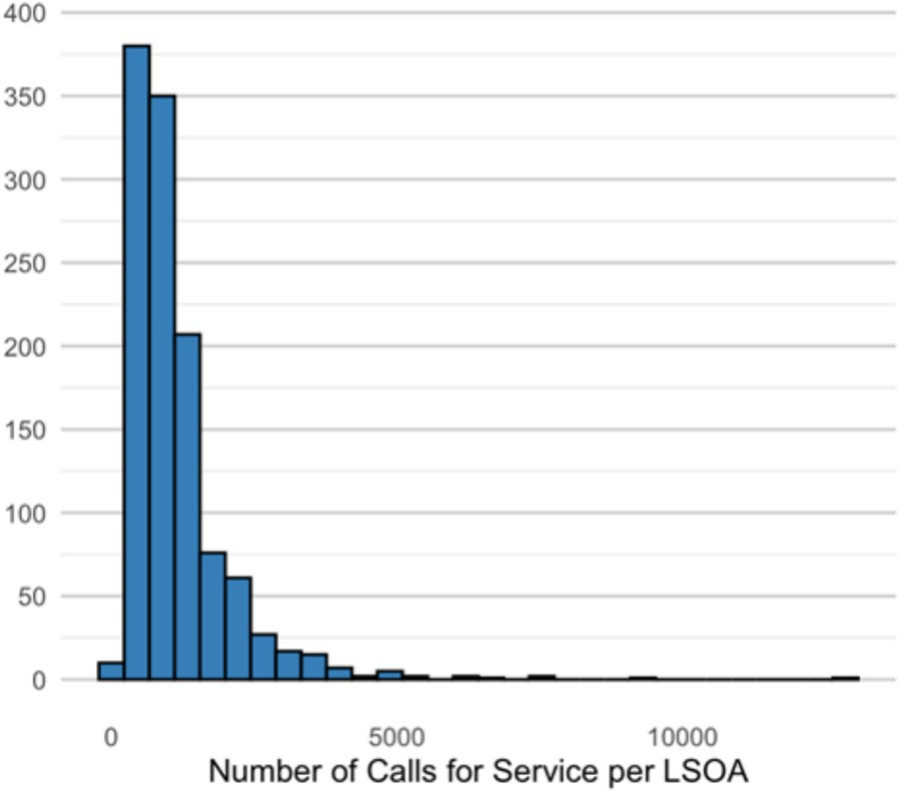
Total number of calls for service per LSOA

**Fig. 2:**
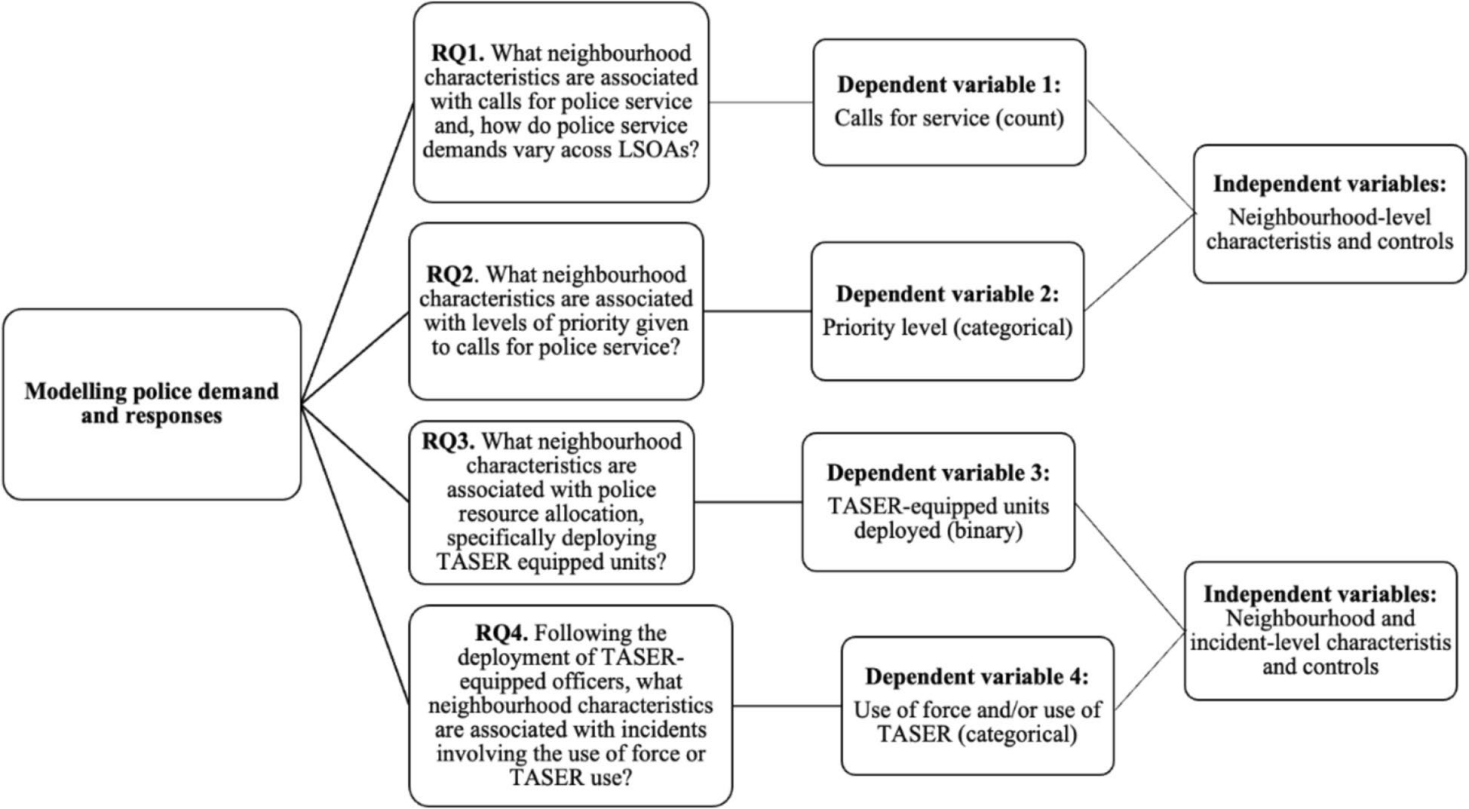
Analytical process for modelling police demand and responses (the results for outcome variables 1, 2, 3 and 4 are in Tables 2, 3, 6 and 7 respectively)

After linking the police administrative datasets, we integrated them with census-based measures at the LSOA level. LSOAs, introduced by the Office for National Statistics following the 2001 Census, are small, standardised geographical areas designed for statistical reporting. In England there are 33,755 LSOAs, each typically containing between 400 and 1200 households, with a resident population ranging from 1000 to 3000 people. The police force area under examination encompasses approximately 1000–1500 LSOAs.^3^ However, due to changes in LSOA classifications over time and inconsistencies in merging data with other sources used in the analysis, some LSOAs were excluded. The final dataset includes data for 1,310,574 observations across 1166 LSOAs, representing 90–95% of the total LSOAs within the force’s operational territory. Figure 1 presents the distribution of calls for service across the 1166 LSOAs and highlights the variability in demand for police resources within the force area. Although call volume is a count variable, the distribution is heavily right-skewed with large values across LSOAs. We applied a log transformation to address this skew, which supported the use of OLS. Model diagnostics indicated that key assumptions were reasonably met. Figure 1 shows the raw distribution, while Figure S1 (in the supplementary materials) shows the log-transformed version, which demonstrates an approximately normal distribution and justifies the modelling approach (Fig. 2).

### Outcome measures

We model the relationship between service demands made by members of the public, subsequent police classification of seriousness of each call, employment of TASER-carrying officers, use of force and their association with neighbourhood-level factors, including area-level mental health (see figure 2) . There are four outcome measures:

1. Total number of calls for service per LSOA.^4^
2. Priority level assigned to calls by call handlers using the National Call Grading System (2005). Under this system, Grade 1 represents an emergency response requiring immediate attendance, Grade 2 indicates a priority response requiring the earliest practicable attendance, Grade 3 involves a scheduled or planned response, and Grade 4 entails a resolution without deployment. Dummy variables were created for Grades 1 and 2, with Grades 3 and 4 combined as the reference category. 
3. Resource allocation decisions based on the nature of the incident as reported by the caller. These decisions include whether the situation warrants the deployment of TASER-equipped units. To capture this, we include a binary variable, where 0 represents no deployment and 1 represents the deployment of TASER-equipped officers.
4. Use of force by responding officers, operationalised using dummy variables to represent three categories: any use of force excluding TASER, TASER use in any mode, and no use of force, which serves as the reference category. Any use of force excluding TASER includes the use of physical force such as restraint tactics, unarmed skills and the use of other equipment, such as batons or irritant sprays. However, it is most often handcuffing.

### Key neighbourhood predictors

- *Black Resident Population:* The ethnic composition of LSOAs is calculated as the percentage of the resident population identifying as Black or Black British, based on 2021 census estimates. A higher percentage indicates greater ethnic diversity or heterogeneity within the LSOAs.
- *Small Area Mental Health Index (SAMHI):* This index, which reflects the mental health needs of the resident population within each LSOA, is constructed using data from multiple sources, including NHS records of mental health-related hospital attendances, prescribing data for antidepressants, Quality and Outcomes Framework statistics on depression, and Department for Work and Pensions data on incapacity benefits and employment support allowance claims related to mental illness (Daras & Barr, 2021). We use the 2019 SAMHI measure, as it falls midway within the study period and predates the COVID-19 pandemic, providing a representative baseline unaffected by the pandemic’s impact on mental health.
- *Concentrated Deprivation:* A census-based measure that captures household deprivation within LSOAs, derived from the 2021 “Households by Deprivation Dimensions” data. It includes four dimensions: education, employment, health, and housing.
- *Residential Turnover:* A measure of residential mobility within LSOAs that is calculated as the percentage of individuals who changed residence within the United Kingdom in the preceding year. Data are sourced from the Migrant Indicator 2021 census dataset.
- *Single-Headed Households:* This census-based measure captures the proportion of lone-parent households with at least one dependent child within each LSOA, based on the Household Composition 2021 census dataset.
- *Response priority & incident threat*: We construct a categorical variable that combines the assigned call grade with a binary indicator of whether the reported incident^5^ was recorded as a violent or non-violent crime. Violent crime is defined as any offence involving physical force or the threat of force against a person, including but not limited to assault, robbery, and sexual offences. Non-violent crime refers to offences that do not involve direct harm or threat to a person, such as theft, criminal damage, and drug-related offences. This variable was then recoded into a series of dummy variables capturing all combinations of call grade and incident type. The resulting categories are: Grade 1–Violent, Grade 1–Non-Violent, Grade 2–Violent, Grade 2–Non-Violent, Grade 3 or 4–Violent, and Grade 3 or 4–Non-Violent, which serves as the reference category.

### Control variables

We include  five additional neighbourhood-level control variables to account for structural and demographic differences across LSOAs. First, we include a census-based measure of the number of residents within each geographical area, which we log-transform to normalise the distribution. Second, we include a measure of crime^6^ using the crime domain index from the 2019 Index of Multiple Deprivation. The crime decile ranks local areas from the highest to the lowest crime prevalence and divides them into ten groups. We reverse the order for ease of interpretation, so 1 indicates areas with the lowest crime rates and 10 represents areas with the highest crime prevalence. Although this data pertains to 2016–2018, it serves as a structural measure of crime within neighbourhoods. Third, we control for the percentage of the LSOA population aged 15–34, as this demographic group is most frequently targeted by police, and fourth, we include the percentage of the population identifying as male. Both measures were derived from the 2021 census. Finally, we include population density, calculated as the number of residents per square kilometre in each LSOA, based on 2021 census estimates.

### Analytical strategy

First, to examine how calls for service vary with neighbourhood structural characteristics (RQ1), we fit two linear regression models.^7^ The first includes the percentage of Black residents, concentrated disadvantage, residential turnover, single-parent households, and crime decile. The second model adds SAMHI. To account for potential confounding factors,  we control for the log-transformed total resident population, the percentage of the population aged 15–34, the percentage of male residents, and population density per square mile in each of our models.

Second, we examine the prioritisation of reported incidents, focusing on how neighbourhood structural characteristics are associated with the assigned opening call grade (RQ2). The same neighbourhood-level factors and controls are included as in the previous step, with mental health prevalence added in the second model. Using multinomial logistic regression, we model call grading^8^ as the dependent variable, with three categories: grade 1 (emergency), grade 2 (priority), and grades 3 and 4 (routine or scheduled responses).

Third, we analyse resource allocation by examining the deployment of TASER-equipped units and their association with neighbourhood characteristics (RQ3a). This stage involves five binary logistic regression models, all using TASER-equipped officer deployment as the dependent variable. Models 1 and 2 include the same neighbourhood-level factors and control variables as in previous models, with SAMHI added in Model 2. Model 3 introduces the Priority Level and Threat variable (a set of dummies combining call grade and incident violence). Model 4 incorporates all predictors into a fully specified model. To answer RQ3b, Model 5 adds an interaction term between deprivation and SAMHI to assess their combined effect on TASER-equipped officer deployment.

Fourth, we narrow the focus to incidents involving TASER-equipped officers to examine the factors associated with use-of-force outcomes, and specifically TASER use (RQ4a). Prior research suggests that the presence of TASER-equipped officers can alter situational dynamics, increasing the likelihood of force being used (Ariel et al., 2019). By restricting the analysis to these incidents, we aim to isolate the neighbourhood- and incident-level factors driving these outcomes. As in the previous stage, we estimate five multinomial logistic regression models. Model 1 includes neighbourhood-level factors and controls. Model 2 adds SAMHI. Model 3 incorporates the Priority Level and Threat variable. Model 4 combines all predictors into a full model, and Model 5 includes an interaction between deprivation and SAMHI (to answer RQ4b).

Finally, we assess multicollinearity using Variance Inflation Factors (VIFs). Most predictors fell below the conventional threshold of 5, though a few exceeded it. In the fully specified models, VIFs for the population aged 15–34 and residential turnover ranged from 5.5 to 9.1—highest in the multinomial model. These suggest moderate collinearity, particularly between age and mobility measures, but none crossed the typical concern threshold of 10 (O’Brien, 2007). Given the theoretical relevance of these variables and model stability, all were retained. Full diagnostics are available on request.

Before presenting the results, it is important to note that we employ a step-wise regression approach, progressively adding covariates to illustrate how key predictors change as additional controls are introduced. This strategy is intended to provide transparency regarding how different factors predict the spatial and social patterning of calls for service, police response grades and use of force. Given the dynamic and context-dependent nature of these factors, no single model can fully encapsulate the complexities of real-time geography. Instead, by presenting a sequence of models, we allow readers to assess the extent to which different variables shape observed patterns. While omitted variable concerns are always present in observational research, our approach helps clarify how various factors relate to one another, without making strong assumptions about causality. This provides a nuanced understanding of police activity and its spatial distribution while also maintaining transparency in our model-building process.

## Results

### Descriptive statistics

Table 1 shows the descriptive statistics of the outcome variables.^9^ The force area received just over 1.3 million calls for service in 2018–2021, Of these 11% were classified as high priority (Grade 1: 15-min response time) by call handlers. Some 35% were graded with the next level of priority (Grade 2: 60-min response time). Officers were dispatched in just over one-third (36%) of the calls for service, with 21% of those cases involving a Taser officer. Force was used in only a small proportion of calls (0.7%) and TASERs were used in incidents associated with just 0.1% of calls. In cases where an officer was dispatched, over half involved a TASER-equipped officer, although the use of TASER remained infrequent, highlighting that TASER deployment does not necessarily correspond to frequent utilisation.

**Table 1:** Breakdown of key variables

	N	Percentage (%)
Calls for Service (All)	1,310,574	100
Call Grade 1	151,953	11.6
Call Grade 2	468,039	35.7
Call Grade 3	184,384	14.1
Call Grade 4	474,915	36.2
Call Grade Unknown	31,283	2.4
Officer(s) Dispatched	474,517	36.2
TASER Officer Dispatched	275,813	21.0
Use of Force (exc. TASER)	8615	0.7%
TASER	918	0.1%

### Multivariate analysis

RQ1: What neighbourhood characteristics are associated with calls for police service?

Table 2 uses Linear regression to model the occurrence of calls for service within LSOAs. In Model 1, where the dependent variable is the log-transformed count of calls for service, key predictors reveal that areas with larger Black populations are associated with an increase in expected calls for service (*b* = 0.06, p < 0.001). Calls for service are also significantly more frequent in neighbourhoods with higher levels of deprivation (*b* = 0.18, p < 0.001), higher crime (*b* = 0.34, p < 0.001), and greater residential turnover (*b* = 0.08, p < 0.001), even after adjusting for baseline population levels, captured through a log-transformed measure of total resident population.

**Table 2:** Linear regression predicting log-transformed calls for service

	Model 1			Model 2		
	*Coefficients*	*CI*	*p*	*Coefficients*	*CI*	*p*
Intercept	− 0.89	− 1.89–0.11	0.080	− 0.93	− 1.92–0.06	0.067
Black Resident Population	0.06	0.03–0.10	< 0.001	0.07	0.04–0.10	< 0.001
Concentrated Deprivation	0.18	0.14–0.21	< 0.001	0.15	0.11–0.19	< 0.001
Residential Turnover	0.08	0.04–0.13	< 0.001	0.09	0.05–0.14	< 0.001
Single-headed households	− 0.02	− 0.06–0.02	0.242	− 0.03	− 0.06–0.01	0.202
Crime decile	0.34	0.30–0.37	< 0.001	0.32	0.29–0.35	< 0.001
Aged 15–34 Resident Population	0.01	− 0.05–0.06	0.826	0.01	− 0.05–0.06	0.758
Male resident population	0.02	− 0.01–0.05	0.249	0.02	− 0.01–0.04	0.257
Population Density	− 0.14	− 0.18– − 0.11	< 0.001	− 0.14	− 0.18– − 0.11	< 0.001
Total Resident Population (log)	1.04	0.91–1.18	< 0.001	1.05	0.91–1.18	< 0.001
SAMHI index 2019				0.05	0.01–0.08	0.005
Observations	1166			1166		
R^2	0.63			0.63		
Adj. R^2	0.63			0.63		

Bold values indicate statistical significance at any of the alpha levels

When mental health prevalence (as measured by SAMHI) is added in Model 2, the results remain largely consistent, with similar coefficient estimates across predictors. Importantly, mental health prevalence emerges as a statistically significant predictor of calls for service (*b* = 0.05, p < 0.01). Interestingly, population density shows a negative association with calls for service in both models, controlling for other variables (*b* = − 0.14, p < 0.001). This suggests that, all else being equal, calls for service are more frequent in less densely populated areas.

RQ2: What neighbourhood characteristics are associated with the levels of priority given to police calls for service?

Table 3 presents the results from multinomial logistic regression models predicting the priority level assigned to calls for service. In Model 1,  priority responses (Grades 1 and 2) tend to originate from neighbourhoods characterised by residential instability, crime and demographic factors. In Model 2, which includes mental health prevalence, Grade 1 responses are more likely in areas with higher proportions of single-headed households (OR = 1.06, p < 0.001), higher crime(OR = 1.08, p < 0.001), larger populations of young people (OR = 1.06, p < 0.001), larger male populations (OR = 1.02, p < 0.001) and higher total resident population (OR = 1.12, p < 0.001). Turning to Grade 2, mental health prevalence is a significant positive predictor (OR = 1.01, p < 0.01), even after controlling for neighbourhood characteristics such as deprivation, crime rates, and residential turnover. Notably, both Grade 1 and Grade 2 responses are negatively associated with population density (OR = 0.97, p < 0.01). The negative association between Grade 1 and Grade 2 responses and densely populated areas suggests a systematic de-prioritisation of incidents in high-demand, highly populated areas due to constrained resources (Bannister et al., 2025).

**Table 3:** Multinomial logistic regression predicting incident response (reference category: all other call grades)

	**Model 1**				**Model 2**		
	*Odds Ratio*	*CI*		*P*	*Odds Ratio*	*CI*	*P*
**Grade 1**							
**Intercept**	0.10	0.08–0.12	** < 0.001**		0.09	0.07–0.12	** < 0.001**
Black Resident Population	0.99	0.98–1.00	**0.003**		0.98	0.97–0.99	** < 0.001**
Concentrated Deprivation	0.98	0.97–0.99	** < 0.001**		1.00	0.99–1.01	0.851
Residential Turnover	0.95	0.94–0.96	** < 0.001**		0.94	0.93–0.95	** < 0.001**
Single-headed households	1.06	1.05–1.07	** < 0.001**		1.06	1.05–1.07	** < 0.001**
Crime decile	1.07	1.06–1.07	** < 0.001**		1.08	1.07–1.09	** < 0.001**
Aged 15–34 Resident Population	1.06	1.04–1.08	** < 0.001**		1.06	1.04–1.08	** < 0.001**
Male resident population	1.02	1.01–1.03	** < 0.001**		1.02	1.01–1.03	** < 0.001**
Population Density	0.96	0.95–0.97	** < 0.001**		0.96	0.95–0.96	** < 0.001**
Total Resident Population (log)	1.12	1.08–1.16	** < 0.001**		1.12	1.09–1.16	** < 0.001**
SAMHI index 2019					0.96	0.95–0.96	** < 0.001**
**Grade 2**							
**Intercept**	1.19	1.01–1.39	**0.033**		1.19	1.02–1.39	**0.030**
Black Resident Population	1.02	1.01–1.02	** < 0.001**		1.02	1.01–1.02	** < 0.001**
Concentrated Deprivation	0.99	0.99–1.00	0.088		0.99	0.98–1.00	**0.007**
Residential Turnover	1.01	1.00–1.02	0.172		1.01	1.00–1.02	0.069
Single-headed households	1.06	1.05–1.06	** < 0.001**		1.06	1.05–1.06	** < 0.001**
Crime decile	1.03	1.03–1.04	** < 0.001**		1.03	1.03–1.04	** < 0.001**
Aged 15–34 Resident Population	1.04	1.03–1.05	** < 0.001**		1.04	1.03–1.05	** < 0.001**
Male resident population	1.02	1.02–1.03	** < 0.001**		1.02	1.02–1.02	** < 0.001**
Population Density	0.97	0.97–0.98	** < 0.001**		0.97	0.97–0.98	** < 0.001**
Total Resident Population (log)	0.93	0.91–0.95	** < 0.001**		0.93	0.91–0.95	** < 0.001**
SAMHI index 2019					1.01	1.00–1.01	**0.007**
N	1,310,574				1,310,574		
R^2^/R^2^ adjusted	0.001/0.001				0.001/0.001		

Bold values indicate statistical significance at any of the alpha levels

To set the scene for the remaining analyses, Tables 4 and 5 show that (a) that TASER-equipped officers are much more likely to be sent to Grades 1 and 2 calls and (b) that use of force (including TASER) is much more likely to result from a response to Grades 1 and 2 calls.

**Table 4:** Conditional distribution of Taser officer dispatched by Call Grade

Taser officer dispatched	Grade 1	Grade 2	Grades 3 and 4	Row Total
No	24,659 (16.1%)	186,864 (39.1%)	641,534 (91.8%)	853,057 (64.1%)
Yes	128,218 (83.9%)	290,763 (60.9%)	57,633 (8.2%)	476,614 (35.9%)
Column Total	152,877 (100%)	477,627 (100%)	699,167 (100%)	1,329,671 (100%)

**Table 5:** Conditional distribution of Taser use and use of force (ex.Taser) by Call Grade

Category	Grade 1	Grade 2	Grades 3 and 4	Row Total
No force used	147,494 (96.5%)	474,011 (99.2%)	698,632 (99.9%)	1,320,137 (99.4%)
Use of force (ex. Taser)	4799 (3.1%)	3321 (0.7%)	496 (0.07%)	8616 (0.7%)
Taser	584 (0.4%)	295 (0.1%)	39 (0.01%)	918 (0.07%)
Column Total	152,877 (100%)	477,627 (100%)	699,167 (100%)	1,329,671 (100%)

RQ3a: What neighbourhood characteristics are associated with the deployment of TASER-equipped units?

Table 6 provides results from binary logistic regression models predicting the likelihood of TASER-equipped officers being dispatched following a call for service. Across all models, neighbourhood characteristics remain consistent predictors. In Models 3 and 4, high-priority calls involving both violent and non-violent crimes are strongly associated with TASER-equipped officer deployments (Grade 1 Violent crime: OR = 2.27, p < 0.001; Grade 1 Non-Violent crime: OR = 2.65 p < 0.001; Grade 2 Violent crime: OR = 2.43, p < 0.001; Grade 2 Non-Violent: OR = 3.08, p < 0.001; Grade 3 and 4 Violent crime: OR = 1.27, p < 0.001).

**Table 6:** Binary logistic regression predicting TASER officer dispatch following a call for service

	Model 1			Model 2			Model 3			Model 4			Model 5		
	*Odds Ratios*	*CI*	*p*	*Odds Ratios*	*CI*	*p*	*Odds Ratios*	*CI*	*p*	*Odds Ratios*	*CI*	*p*	*Odds Ratios*	*CI*	*p*
**Intercept**	0.42	0.35–0.50	** < 0.001**	0.42	0.35–0.50	** < 0.001**	0.27	0.22–0.34	** < 0.001**	0.27	0.22–0.34	** < 0.001**	0.28	0.23–0.35	** < 0.001**
Black Resident Population	0.95	0.94–0.96	** < 0.001**	0.95	0.95–0.96	** < 0.001**	0.94	0.94–0.95	** < 0.001**	0.95	0.94–0.95	** < 0.001**	0.94	0.93–0.95	** < 0.001**
Concentrated Deprivation	1.03	1.02–1.03	** < 0.001**	1.02	1.01–1.03	** < 0.001**	1.04	1.03–1.05	** < 0.001**	1.03	1.02–1.04	** < 0.001**	1.06	1.04–1.07	** < 0.001**
Residential Instability	1.00	0.99–1.01	0.403	1.01	1.00–1.02	0.219	1.03	1.02–1.05	** < 0.001**	1.04	1.03–1.05	** < 0.001**	1.03	1.01–1.04	** < 0.001**
Single-Headed Households	1.07	1.06–1.08	** < 0.001**	1.07	1.06–1.08	** < 0.001**	1.02	1.01–1.03	** < 0.001**	1.02	1.01–1.03	** < 0.001**	1.02	1.01–1.03	** < 0.001**
Crime Decile	1.00	0.99–1.00	0.100	0.99	0.99–1.00	**0.021**	0.96	0.96–0.97	** < 0.001**	0.96	0.95–0.97	** < 0.001**	0.96	0.95–0.96	** < 0.001**
Aged 15–34 Resident Population	1.02	1.01–1.03	**0.004**	1.02	1.01–1.03	**0.003**	0.98	0.96–0.99	** < 0.001**	0.98	0.96–0.99	** < 0.001**	0.97	0.96–0.99	** < 0.001**
Male Resident Population	1.01	1.01–1.02	** < 0.001**	1.01	1.01–1.02	** < 0.001**	1.00	1.00–1.01	0.282	1.00	1.00–1.01	0.293	1.01	1.00–1.01	0.080
Population Density	1.05	1.04–1.05	** < 0.001**	1.05	1.04–1.05	** < 0.001**	1.06	1.05–1.06	** < 0.001**	1.06	1.05–1.07	** < 0.001**	1.06	1.05–1.07	** < 0.001**
Total Resident Population (log)	0.94	0.92–0.96	** < 0.001**	0.94	0.92–0.96	** < 0.001**	0.92	0.89–0.94	** < 0.001**	0.92	0.89–0.94	** < 0.001**	0.92	0.89–0.94	** < 0.001**
SAMHI index 2019				1.01	1.00–1.01	**0.017**				1.01	1.00–1.02	**0.001**	1.01	1.00–1.02	**0.010**
GRADE1 Violent Crime							2.27	2.25–2.28	** < 0.001**	2.27	2.25–2.28	** < 0.001**	2.26	2.25–2.28	** < 0.001**
GRADE1 Non-Violent							2.65	2.64–2.67	** < 0.001**	2.66	2.64–2.67	** < 0.001**	2.66	2.64––2.67	** < 0.001**
GRADE2 Violent Crime							2.43	2.42–2.44	** < 0.001**	2.43	2.42–2.44	** < 0.001**	2.43	2.42–2.44	** < 0.001**
GRADE2 Non-Violent							3.08	3.06–3.10	** < 0.001**	3.08	3.06–3.10	** < 0.001**	3.08	3.06–3.10	** < 0.001**
GRADE3 + 4 Violent Crime							1.27	1.26–1.27	** < 0.001**	1.27	1.26–1.27	** < 0.001**	1.27	1.26–1.27	** < 0.001**
Deprivation x SAMHI													0.98	0.97–0.98	** < 0.001**
Observations	1,310,574			1,310,574			1,310,574			1,310,574			1,310,574		
R^2^ Tjur	0.002			0.002			0.257			0.257			0.257		

Bold values indicate statistical significance at any of the alpha levels

In Model 4, even after controlling for priority grade and the incident type, neighbourhood characteristics such as concentrated deprivation (OR = 1.03, p < 0.001), single-headed households (OR = 1.02, p < 0.001) population density (OR = 1.06, p < 0.001), and mental health prevalence (OR = 1.01, p = 0.001) are significant positive predictors. These findings suggest that, beyond the clustering of high-priority responses and incident types, certain neighbourhood characteristics seem to contribute independently to the likelihood of TASER-equipped deployments.

RQ3b: Do mental health and deprivation interact to predict the dispatch of TASER-equipped units?

In Model 5, we include interaction effects between deprivation and mental health. Contrary to expectations, we find a negative interaction between these factors and the likelihood of TASER officers being dispatched following a call for service (OR = 0.98, p < 0.001). This suggests that calls for service from deprived areas with a higher prevalence of mental healths need are less likely to result in the deployment of a TASER-equipped officer, compared to calls for service from deprived areas with a lower prevalence of mental health issues. We return to this finding in the limitations and future research section at the end of the paper.

RQ4a: Following the deployment of TASER-equipped officers, what neighbourhood characteristics are associated with incidents involving use of force or TASER use?

Table 7 uses multinomial logistic regression to examine the use of force (excluding TASER) and TASER use, focusing on calls involving TASER-equipped officers. In Model 2, mental health prevalence is introduced as a predictor. Adding this variable makes concentrated deprivation a significant negative predictor of both use of force ( OR = 0.88, p < 0.001) and TASER use (OR = 0.86, p < 0.05). Additionally, larger Black populations become significant positive predictors of TASER use (OR = 1.13, p < 0.05). In Models 3 and 4, Grade 1 Violent and Non-Violent, Grade 2 Violent and Non-Violent, and Grade 3 and 4 Violent Crime are incorporated into the analysis. Combined, priority levels and incident types, whether violent or non-violent, strongly predict the use of force and TASER deployment.

**Table 7:** Multinomial logistic regression predicting use of force/TASER use following dispatch of TASER units (reference category: no use of force)

	Model 1		Model 2				Model 3			Model 4			Model 5		
	*Odds Ratio*	*CI*	*P*	*Odds Ratio*	*CI*	*P*	*Odds Ratio*	*CI*	*P*	*Odds Ratio*	*CI*	*P*	*Odds Ratio*	*CI*	*P*
UoF (ex. TASER)															
**Intercept**	0.02	0.01–0.05	** < 0.001**	0.02	0.01–0.06	** < 0.001**	0.01	0.00–0.03	** < 0.001**	0.01	0.00–0.03	** < 0.001**	0.01	0.01–0.04	** < 0.001**
Black Resident Population	0.98	0.95–1.02	0.419	1.03	0.99–1.07	0.193	0.99	0.96–1.03	0.717	1.03	0.99–1.08	0.102	1.02	0.98–1.06	0.291
Concentrated Deprivation	0.97	0.93–1.02	0.226	0.88	0.84–0.93	** < 0.001**	0.96	0.92–1.01	0.090	0.87	0.83–0.92	** < 0.001**	0.92	0.87–0.97	**0.001**
Residential Instability	1.00	0.94–1.05	0.884	1.04	0.99–1.10	0.147	1.04	0.98–1.10	0.199	1.08	1.02–1.15	**0.006**	1.05	0.99–1.11	0.112
Single-headed households	1.01	0.97–1.05	0.609	1.00	0.96–1.04	0.997	0.98	0.94–1.02	0.365	0.97	0.93–1.01	0.179	0.96	0.92–1.01	0.090
Crime decile	1.07	1.04–1.11	** < 0.001**	1.02	0.98–1.05	0.413	1.06	1.02–1.10	**0.001**	1.00	0.97–1.04	0.810	1.00	0.96–1.04	0.941
Aged 15–34 Resident Population	1.17	1.10–1.24	** < 0.001**	1.18	1.11–1.26	** < 0.001**	1.13	1.06–1.20	** < 0.001**	1.15	1.08–1.23	** < 0.001**	1.16	1.09–1.24	** < 0.001**
Male resident population	1.02	0.99–1.05	0.171	1.02	0.99–1.05	0.241	1.02	0.99–1.05	0.189	1.02	0.99–1.05	0.272	1.02	0.99–1.06	0.178
Population Density	1.03	0.99–1.06	0.113	1.04	1.01–1.08	**0.008**	0.98	0.95–1.01	0.213	1.00	0.96–1.03	0.834	1.00	0.97–1.03	0.850
Total Resident Population (log)	1.03	0.89–1.18	0.701	1.02	0.88–1.18	0.781	1.01	0.88–1.16	0.897	1.01	0.88–1.17	0.880	0.97	0.84–1.12	0.668
SAMHI index 2019				1.20	1.16–1.25	** < 0.001**				1.20	1.16–1.25	** < 0.001**	1.20	1.15–1.24	** < 0.001**
GRADE 1 Violent Crime							1.51	1.46–1.55	** < 0.001**	1.51	1.46–1.55	** < 0.001**	1.50	1.46–1.55	** < 0.001**
GRADE 1 Non-Violent							1.40	1.35–1.46	** < 0.001**	1.41	1.35–1.47	** < 0.001**	1.40	1.35–1.47	** < 0.001**
GRADE 2 Violent							1.29	1.23–1.35	** < 0.001**	1.29	1.23–1.34	** < 0.001**	1.28	1.23–1.34	** < 0.001**
GRADE 2 Non-Violent							1.15	1.07–1.23	** < 0.001**	1.15	1.07–1.23	** < 0.001**	1.14	1.07–1.23	** < 0.001**
GRADE 3 + 4 Violent Crime							1.05	0.99–1.11	0.100	1.05	0.99–1.11	0.106	1.05	0.99–1.11	0.117
Deprivation x SAMHI													0.94	0.92–0.97	** < 0.001**
TASER															
**Intercept**	0.00	0.00–0.05	** < 0.001**	0.00	0.00–0.05	** < 0.001**	0.00	0.00–0.02	** < 0.001**	0.00	0.00–0.02	** < 0.001**	0.00	0.00–0.03	** < 0.001**
Black Resident Population	1.09	0.98–1.21	0.105	1.13	1.01–1.26	**0.027**	1.10	0.99–1.22	0.074	1.14	1.02–1.27	**0.018**	1.13	1.01–1.25	**0.033**
Concentrated Deprivation	0.93	0.83–1.05	0.233	0.86	0.75–0.98	**0.021**	0.92	0.82–1.04	0.180	0.85	0.74–0.97	**0.015**	0.91	0.78–1.05	0.195
Residential Instability	0.88	0.75–1.05	0.148	0.92	0.77–1.09	0.347	0.92	0.78–1.09	0.354	0.96	0.80–1.14	0.611	0.92	0.77–1.10	0.385
Single-headed households	1.12	1.00–1.25	0.061	1.11	0.99–1.24	0.086	1.09	0.97–1.22	0.154	1.08	0.96–1.21	0.199	1.07	0.95–1.20	0.288
Crime decile	1.00	0.91–1.10	0.996	0.95	0.86–1.06	0.379	0.99	0.89–1.09	0.785	0.94	0.85–1.05	0.268	0.93	0.84–1.04	0.203
Aged 15–34 Resident Population	1.13	0.94–1.37	0.195	1.14	0.94–1.38	0.170	1.10	0.91–1.32	0.346	1.11	0.92–1.34	0.288	1.11	0.92–1.35	0.276
Male resident population	1.03	0.94–1.13	0.474	1.03	0.94–1.13	0.518	1.03	0.94–1.13	0.479	1.03	0.94–1.12	0.552	1.03	0.94–1.13	0.473
Population Density	1.06	0.97–1.16	0.189	1.08	0.99–1.18	0.098	1.02	0.93–1.11	0.730	1.03	0.94–1.13	0.513	1.03	0.95–1.13	0.460
Total Resident Population (log)	1.03	0.69–1.54	0.890	1.03	0.68–1.55	0.889	1.00	0.67–1.50	0.997	1.04	0.69–1.56	0.863	0.99	0.65–1.49	0.955
SAMHI index 2019				1.17	1.05–1.31	**0.004**				1.17	1.05–1.31	**0.005**	1.16	1.04–1.30	**0.007**
GRADE 1 Violent Crime							1.57	1.43–1.72	** < 0.001**	1.57	1.43–1.73	** < 0.001**	1.56	1.42–1.71	** < 0.001**
GRADE 1 Non-Violent							1.55	1.36–1.77	** < 0.001**	1.56	1.36–1.78	** < 0.001**	1.54	1.35–1.76	** < 0.001**
GRADE 2 Violent Crime							1.32	1.15–1.52	** < 0.001**	1.32	1.15–1.52	** < 0.001**	1.31	1.14–1.50	** < 0.001**
GRADE 2 Non-Violent							1.12	0.89–1.41	0.314	1.13	0.90–1.43	0.286	1.11	0.89–1.39	0.361
GRADE 3 + 4 Violent Crime							0.99	0.81–1.22	0.926	0.99	0.81–1.22	0.953	0.97	0.79–1.20	0.809
Deprivation x SAMHI													0.94	0.87–1.01	0.080
N	275,813		275,813				275,813			275,813				275,813	
R^2^/R^2^ adjusted	0.005/0.005		0.006/0.006				0.056/0.056			0.058/0.058			0.058/0.058		

Bold values indicate statistical significance at any of the alpha levels

For Grade 1 Violent Crime calls, the odds of use of force increase (OR = 1.51, p < 0.001), as do the odds of TASER use (OR = 1.57, p < 0.001). Similar patterns are observed for Grade 1 Non-Violent Crime, with increased odds of use of force (OR = 1.41, p < 0.001) and TASER (OR = 1.56, p < 0.001). Grade 2 Violent crime calls show similar patterns for both force (OR = 1.29, p < 0.001) and TASER use (OR = 1.32, p < 0.001). The increased in odds of force is shown when looking at Grade 2 Non-Violent crime (OR = 1.15, p < 0.01).??

In Table 7, a negative partial correlation between concentrated deprivation and both police use of force and TASER use is evident across all models and warrants further discussion. While this might seem counterintuitive, it does not mean deprivation is unimportant. On the contrary, deprivation seems to be central to the overall process. Calls for service are heavily concentrated in disadvantaged areas with high mental health needs, placing these neighbourhoods at the front line of police response. That demand is then shaped by operational decisions—such as response grading and TASER deployment—that reinforce patterns of police presence, even if, at this stage in the process, deprivation is negatively correlated with use of force.

RQ4b: Do mental health and deprivation interact to predict incidents that involve the use of force and/or TASER use?

In the final model, we introduce interaction effects between concentrated deprivation and mental health. Again, the interaction effect between deprivation and mental health is negative, and this is for both the use of force (OR = 0.94, p < 0.001) and TASER use (OR = 0.94, p > 0.05), albeit only the first interaction effect is statistically significant. As with RQ3b, we turn to this in the limitations and future research section.

Overall, then, if use of force incidents arising from calls for service were evenly distributed across neighbourhoods, we would expect a corresponding uniformity in each preceding decision point—from the public’s initial call to the dispatch of TASER-equipped officers to the incident itself. But our study did not find this kind of uniformity. Instead, our findings suggest a cumulative layering effect, with policing demand and use of force rooted in structural inequalities, operational decisions and repeated exposure to police presence in disadvantaged areas. We found that police demand and response clustered in neighbourhoods with already high levels of poor mental health, crime, structural disadvantage and residential instability. These areas also had younger populations and a larger proportion of Black or Black British residents. Crucially, the relationship between mental health prevalence and decisions made at each stage of the process remained consistent even after controlling for other neighbourhood characteristics. This suggests that mental health challenges play a distinct role in shaping police activity, independent of other structural factors.

## Discussion and conclusions

Despite extensive research on police use of force, the role of calls for service in predicting *where*, *when* and *against whom* force is applied has received comparatively little attention. Force can only be used where officers are physically present, and while many deployments result from public calls, few studies have examined how these calls are geographically distributed, how they inform operational decisions like priority grading and officer allocation, and how they function as an unseen driver of neighbourhood disparities in police use of force.

Drawing on routine activity theory (Cohen & Felson, 1979) and the social ecology of crime and deprivation (Shaw & McKay, 1942; Sampson & Wilson, 1995), our findings show that police—at least in one English police force—are drawn to high-demand areas where structural disadvantage and mental health distress create opportunities for intervention. These context characteristics predicted not only *where* police go but also *how* they respond—including whether TASER-equipped officers are dispatched. What emerged was a layered structure of disparity: disadvantaged neighbourhoods generated more calls, received higher priority gradings, and were more likely to have TASER-equipped officers deployed—culminating in a higher likelihood of force being used. This process unfolded not through any single act of discretion, but via a chain of decisions made by institutions and publics alike.

This dynamic is rooted in routine activity theory, which emphasises how institutional routines shape the opportunity structures within which force encounters occur (Lum, 2011; Miller et al., 2024). It also echoes key insights from the social ecology tradition, which links structural disadvantage and community disorganisation to concentrated policing and enforcement (Nouri, 2021; Sampson & Wilson, 1995). Together, these frameworks suggest that use of force is not merely the outcome of individual choices, it is also the product of institutional logics embedded in structurally unequal environments.

Although our study does not directly examine disproportionalities in use of force—such as racial disparities—our findings contributes to this broader debate, illustrating how the effect of disadvantage on police activity becomes compounded through routine, sequential decision-making. Our results align with the cumulative disadvantage framework advanced by Guarnera et al. (2024), which posits that disparities arise not from isolated acts of bias but from institutional processes that unfold over time. In our case, the sequence began with a public decision to call the police; call handlers assigned a priority level, shaping the urgency and type of response; a further decision was made about whether to dispatch a TASER-equipped officer; and finally, the attending officer decided whether and how to use force. At each stage, neighbourhood-level characteristics—particularly deprivation and mental health need—predicted the decisions made. The result was a pattern of geographic inequality in force application that arises through institutional routines, even in the explicit absence of overt bias at any one point.

Our findings on mental health deserve specific comment. They suggest that calls for service reflect systemic gaps in public health and social care provision—placing police in the role of de facto crisis responders—with the use of force reflecting not a direct response to crime but a consequence of institutional overreach and policy failure elsewhere (Kyprianides & Bradford, 2024, 2025). Addressing these upstream drivers requires more than procedural reform, we would argue; it demands a broader rethinking of how public services allocate responsibility and risk. Two policy implications follow from this. First, improved mental health services in high-need areas could reduce the reliance on police as frontline responders, thereby lowering the risk of force. Second, call-handling protocols could be refined to better assess mental health need and triage appropriate non-police responses where feasible.

### Limitations

Several limitations should be acknowledged. First, around half of all recorded use of force incidents occurred outside the calls for service framework—highlighting the need for further research on proactive and discretionary policing. Second, our analysis of call priority relies on the initial grading by call handlers and does not account for subsequent reclassifications once officers arrive. Third, while we document strong associations between neighbourhood conditions and policing decisions, we do not make causal claims—our findings are exploratory and should be interpreted as such. Fourth, there is a risk of ecological fallacy: neighbourhood-level patterns do not necessarily reflect individual experiences.

Finally, we have presented a pattern of compounding effects that unfold in a broadly linear fashion. This reflects both an effort to simplify what is likely to be an iterative and non-recursive process and, to some extent, a real feature of how police responses ‘escalate.’ Our findings suggest that in areas with greater mental health challenges, at every stage—from call volume to call grading to officer deployment—the likelihood of TASER being used is elevated. However, in practice, this process is unlikely to be entirely linear. Decision-making by call handlers, resource constraints and officer discretion all introduce the potential for feedback loops and divergence from a strictly sequential model. As always, statistical modelling provides an approximation of complex social processes, illuminating some aspects while inevitably obscuring others.

### Future research

We see two key avenues for future lines of enquiry. First, our findings on the role of mental health warrant closer examination. As outlined in our theoretical framing, we approached the interaction between mental health need and deprivation as exploratory, recognising that the interaction effect could plausibly go in one of two directions. We found that the link between mental health need and both TASER-equipped officer deployment and use of force was weaker in more deprived neighbourhoods than it was in less deprived ones. This is consistent with one of the mechanisms we proposed: that in less deprived areas, signs of mental distress may be perceived as more anomalous or disruptive, prompting greater public concern and more escalated police responses. By contrast, similar behaviours in more deprived contexts may be viewed as more routine, and thus less likely to elicit a heightened police response.

Further research is needed to replicate this finding and to empirically investigate the mechanisms that might underpin it—including whether officers and call handlers interpret mental distress as more problematic in communities less accustomed to such behaviour, and respond more readily (or more coercively) out of a perceived obligation to maintain public order or reassure local residents. Scholars should also examine more closely what area-level indicators of mental health capture in this context. While we interpret the observed association as reflecting officer responses to individual-level mental health distress, this remains an assumption. The aggregate measure may also reflect broader neighbourhood dynamics or institutional patterns of service demand.

Second, longitudinal studies should explore how repeated exposure to high-intensity calls shapes both community–police relations and officer decision-making over time. Frequent encounters involving coercive tactics—particularly where concentrated in specific areas—may erode public trust, reinforce perceptions of over-policing, and influence residents’ willingness to report incidents or cooperate with police. For officers, regular exposure to high-stakes or ambiguous situations may foster desensitisation, risk aversion or reliance on heuristics that shape future responses. Future work could examine whether such cumulative exposure leads to behavioural adaptation, emotional burnout or shifts in tactical preferences—especially in neighbourhoods marked by persistent vulnerability or high demand.

Complementary qualitative research should also provide essential insight into how these dynamics are experienced in practice, on the ground. Interviews or ethnographic work with officers, call handlers and residents could illuminate how decisions around call grading, unit deployment and escalation are influenced by prior experience, organisational context and community expectations. In particular, researchers could attend to how perceptions of fairness, risk and legitimacy evolve among both frontline practitioners and the communities they serve under sustained conditions of operational pressure.

### Final words

To conclude, our findings highlight the need for a holistic, process-based understanding of police use of force. By tracing how calls for service trigger a sequence of decisions—from incident prioritisation, TASER-equipped deployments, to the application of force—we show how structural disadvantage, mental health distress and institutional routines combine to concentrate force encounters in already disadvantaged neighbourhoods. This challenges the idea that force is simply a reactive response to criminality. Our study shows that operational choices actively shape the geography of police intervention and reproduce spatial inequalities.

For policymakers, the implication is clear: disparities in policing are not always traceable to individual moments of bias or misconduct. Often, they are the result of routine decisions made within structurally constrained systems. Reform efforts must therefore address not just what happens at the point of contact, but the upstream decisions and institutional logics that bring officers into contact with particular communities in the first place.

Finally, the cumulative disadvantage framework offers a powerful lens for understanding how seemingly neutral institutional processes can entrench inequality (Guarnera et al., 2024). To produce more equitable policing outcomes, we must reform not only frontline practices, but the broader systems through which public safety is delivered, especially in communities where mental health need and structural disadvantage intersect. Understanding police use of force as the outcome of layered institutional processes, rather than isolated incidents, opens new pathways for designing fairer, more responsive models of public safety.

## Supplementary Information

Additional file 1.

## Data Availability

Unfortunately, they are not publicly available because of the agreement put in place with the English police force.

## Footnotes

1 While calls for service constitute a major source of police demand, they represent only a portion of the overall picture, which also encompasses proactive policing, internal obligations (such as administrative tasks) and “failure demand” resulting from incomplete or incorrect actions (Laufs et al., 2021). Both reactive and proactive police activities are distributed geographically based on these diverse demands—and on how officers choose to respond.

2 The data for this study was originally collected as part of the TASER and Social, Ethnic, and Racial Disparities research project, commissioned by the College of Policing and funded by the National Police Chiefs' Council and the Mayor's Office for Policing and Crime.

3 To anonymise the police force, we do not report the precise number of LSOAs.

4 Although a call for service may involve emergency requests directed to fire and rescue or ambulance services, we focus on calls made to the police service.

5 Incident type refers to the category assigned by the call handler at the time of the call and is drawn from over 200 possible classifications. We do not include incident type as a separate predictor in the models due to the broad distribution of violent incidents across all call grades.

6 To assess the robustness of our findings, we re-ran all models using police-recorded crime rates (from data.police.uk, 2017–2020) as an alternative specification. The results remain largely consistent and are presented in the supplementary materials.

7 We tested the two models using three different specifications: (1) a linear model of the calls for service rate per 1,000 residents; (2) a linear model of log-transformed calls for service rate; and (3) a negative binomial model. AIC comparisons indicated that both linear models provided better fit than the negative binomial model. The linear rate model had an AIC of 17,102.76, compared to 17,183.35 for the negative binomial model. The log-transformed linear model offered substantially better fit than both (AIC = 1,186.87). BIC values followed the same pattern, with the log-transformed OLS model (BIC = 1,247.61) outperforming both the rate-based OLS model (BIC = 17,163.50) and the negative binomial model (BIC = 17,244.08). Based on these results, we present the OLS model using the log-transformed dependent variable as our main specification.

8 Calls with unknown grades (N = 31,682, 2.40%) are included in the reference category. Sensitivity tests were conducted to examine the implications of this approach, with the results discussed in the supplementary materials.

9 Full descriptive statistics and the correlation matrix for all outcome and predictor variables are provided in the supplementary materials, including commentary.
