# Supplementary material for: The compounding effect: how neighbourhood dynamics shape police deployment and use of force
Source: Crime Sci. 2025 Oct 2;14(1):15. doi: 10.1186/s40163-025-00258-6 (PMC12491367; doi:10.1186/s40163-025-00258-6)
Supplement: Supplementary file 1 — Additional file 1. [file 40163_2025_258_MOESM1_ESM.docx]

SUPPORTING INFORMATION FOR

“THE COMPOUNDING EFFECT: HOW NEIGHBOURHOOD DYNAMICS SHAPE DEPLOYMENT AND POLICE USE OF FORCE”

CONTENTS

S1: DESCRIPTIVE STATISTICS AND CORRELATIONS

S2: SENSITIVITY TESTS

**Supplement S1. Descriptive statistics and correlations**

The percentage of Black/Black British residents ranges from 0% to a maximum of 13.1% (Table S1). Furthermore, the mean percentage of residents experiencing concentrated deprivation across LSOAs is 3%. Similar to the distribution of Black/Black British residents, concentrated deprivation is absent from some LSOAs, as indicated by a minimum value of 0%. Specifically, for residential turnover, a standard deviation of 4.7% highlights significant variation in residential mobility among LSOAs, with some areas showing very low levels of mobility whilst others experience much higher rates of residential turnover. Additionally, single-headed households account for an average of 5.7% of all households, suggesting that, on average, 5.7% of households in each LSOA are led by a single parent responsible for at least one dependent child.

As expected, most variables demonstrate moderate to strong correlations, suggesting that calls for service, police activity, crime rates, ethnic composition, socioeconomic challenges, the presence of young people, higher proportion of male residents and mental health are closely interconnected. Calls for service tend to be higher in areas with a larger Black/Black British resident population (r=.50), in more deprived areas (r=.45), areas where there is residential turnover (r=.39), single-headed households (r=.28), higher crime rates (r=.50), areas with a larger presence of young people (r=.43), higher population of Male residents (r=.18), and areas where there are more people experiencing mental health issues(r=.25). Similarly, Grade 1 calls—the highest priority calls—tended to be more frequent in areas with a larger Black/Black British resident population (r=.45), in more deprived areas (r=.42), residential turnover (r=.32), single-headed households (r=.29), high crime areas (r=.48), larger population of young people (r=.39), larger male population (r=.16), and areas with mental health (r=.23).

The deployment of Taser-equipped officers also follows similar patterns. Taser-equipped officers are more likely to be dispatched to calls for service in areas with a larger Black/Black British resident population (r=.53), higher levels of deprivation (r=.55), residential turnover (r=.39), more single-headed households (r=.38), high crime rates (r=.55), larger populations of young people (r=.47), higher proportions of male residents (r=.18), and areas where there are more people with mental health issues (r=.31). Taser use tends to occur more in areas with larger Black/Black British resident population (r=.40), in more deprived areas (r=.36), residential turnover (r=.25), single-headed households (r=.28), higher crime rates (r=.34), larger populations of young people (r=.35), larger male population (r=.14), and areas where there are more people with mental health issues (r=.21).

**Supplement S2. Sensitivity Tests**

To ensure the robustness of our findings, we undertook a number of sensitivity tests. First, we explored the pattern of missing call grades (N = 31,682) by examining the relationship between the presence of missing grades and whether an officer was dispatched. The results indicate that missing call grades were uncommon when an officer was dispatched, occurring in less than 1% of cases (N = 4,449). In contrast, 3.2% of calls resolved without officer deployment did not include call grade information (N = 27,233). This discrepancy could be attributed to abandoned calls or administrative logging errors. To evaluate the robustness of our results, we estimated models that alternately excluded and included observations with missing call grades in the reference category when modelling call grades as the outcome. The reference category was deemed most appropriate as it represents instances where officers were not deployed. Across both specifications, the findings remained consistent, suggesting that missing call grades do not significantly impact the main results.

Second, while LSOAs provide a consistent population base, we considered whether modelling the dependent variable as a raw count or per 1,000 residents might yield different results. To assess this, we conducted a sensitivity test using calls for service per 1,000 residents. The results remained largely consistent with the main model, showing similar positive associations with key predictors.

Third, we tested for potential temporal effects, as the data overlap with the COVID-19 period, which may have influenced both call patterns and officer deployment. We focused on call grades as the outcome for this sensitivity analysis because time is captured at the individual level, and aggregating the data to the LSOA level for the count of calls as the outcome would obscure temporal variation. We created three variables representing the key national lockdown periods^^[[1]](#footnote-1)^^ and included them in the fully specified models. This approach allowed us to capture the potential impact of each lockdown phase on prioritisation given to calls. The results indicate that the inclusion of these lockdown variables did not significantly alter the key findings.^^[[2]](#footnote-2)^^

Finally, to assess the robustness of our findings we re-estimated the model using a set of dummy variables with decile 1 (most deprived) as the reference category. This approach allowed us to assess whether a linear ordinal specification was appropriate. The results revealed a generally increasing pattern across deciles in the earlier models (predicting calls for service), consistent with the linear trend assumed in the ordinal specification. However, this pattern becomes less apparent in the later models. All other explanatory variables and controls remain largely unchanged. Tables S2 to S5 present the results of these alternative specifications.

**Table S1:** Descriptive statistics and correlation matrix

|  | N | Mean | SD | Min. | Max. | 1 | 2 | 3 | 4 | 5 | 6 | 7 | 8 | 9 | 10 | 11 | 12 | 13 | 14 | 15 | 16 | 17 | 18 |
| --- | --- | --- | --- | --- | --- | --- | --- | --- | --- | --- | --- | --- | --- | --- | --- | --- | --- | --- | --- | --- | --- | --- | --- |
| Calls for Service (1) | 1166 | 1140.4 | 962.4 | 165 | 13016 | 1 |  |  |  |  |  |  |  |  |  |  |  |  |  |  |  |  |  |
| Grade 1 (2) | 1166 | 131.1 | 124.7 | 7 | 1286 | 0.91 | 1 |  |  |  |  |  |  |  |  |  |  |  |  |  |  |  |  |
| Grade 2 (3) | 1166 | 409.6 | 382.2 | 54 | 5470 | 0.98 | 0.88 | 1 |  |  |  |  |  |  |  |  |  |  |  |  |  |  |  |
| Grade 3 (4) | 1166 | 158.5 | 135.5 | 14 | 1929 | 0.96 | 0.84 | 0.95 | 1 |  |  |  |  |  |  |  |  |  |  |  |  |  |  |
| Grade 4 (5) | 1166 | 413.9 | 317.9 | 57 | 3859 | 0.97 | 0.85 | 0.92 | 0.92 | 1 |  |  |  |  |  |  |  |  |  |  |  |  |  |
| Dispatched (6) | 1166 | 408.8 | 341.6 | 58 | 4649 | 0.95 | 0.92 | 0.95 | 0.92 | 0.90 | 1 |  |  |  |  |  |  |  |  |  |  |  |  |
| Taser Officer Dispatched (7) | 1166 | 237.8 | 196 | 17 | 2586 | 0.94 | 0.91 | 0.94 | 0.92 | 0.89 | 0.99 | 1 |  |  |  |  |  |  |  |  |  |  |  |
| Use of Force excluding Taser (8) | 1166 | 7.39 | 12.1 | 0 | 175 | 0.74 | 0.66 | 0.77 | 0.75 | 0.68 | 0.81 | 0.80 | 1 |  |  |  |  |  |  |  |  |  |  |
| TASER (9) | 1166 | 0.79 | 1.35 | 0 | 12 | 0.56 | 0.52 | 0.58 | 0.57 | 0.52 | 0.62 | 0.62 | 0.63 | 1 |  |  |  |  |  |  |  |  |  |
| Black/Black British Resident Population % (10) | 1166 | 1.32 | 1.65 | 0 | 13.1 | 0.50 | 0.45 | 0.51 | 0.50 | 0.47 | 0.53 | 0.53 | 0.44 | 0.40 | 1 |  |  |  |  |  |  |  |  |
| Concentrated Deprivation % (11) | 1166 | 3.04 | 2.64 | 0 | 16.4 | 0.45 | 0.42 | 0.45 | 0.46 | 0.43 | 0.52 | 0.55 | 0.34 | 0.36 | 0.41 | 1 |  |  |  |  |  |  |  |
| Residential turnover (12) | 1166 | 9.58 | 4.77 | 3.84 | 59.6 | 0.39 | 0.32 | 0.39 | 0.39 | 0.37 | 0.39 | 0.39 | 0.41 | 0.25 | 0.46 | 0.06 | 1 |  |  |  |  |  |  |
| Single-headed Households % (13) | 1166 | 9.47 | 4.36 | 2.48 | 29.7 | 0.28 | 0.29 | 0.29 | 0.30 | 0.24 | 0.36 | 0.38 | 0.21 | 0.28 | 0.32 | 0.73 | -0.10 | 1 |  |  |  |  |  |
| Crime Decile (14) | 1166 | 5.43 | 2.85 | 1 | 10 | 0.50 | 0.48 | 0.49 | 0.51 | 0.48 | 0.54 | 0.55 | 0.37 | 0.34 | 0.47 | 0.61 | 0.17 | 0.51 | 1 |  |  |  |  |
| Aged 15-34 Resident population % (15) | 1166 | 23.3 | 8.78 | 7.27 | 84.2 | 0.43 | 0.39 | 0.44 | 0.45 | 0.39 | 0.46 | 0.47 | 0.45 | 0.35 | 0.66 | 0.34 | 0.75 | 0.28 | 0.45 | 1 |  |  |  |
| Male Resident Population % (16) | 1166 | 49 | 2.03 | 42.7 | 69.4 | 0.18 | 0.16 | 0.18 | 0.18 | 0.16 | 0.18 | 0.18 | 0.19 | 0.14 | 0.39 | -0.04 | 0.39 | -0.13 | 0.09 | 0.47 | 1 |  |  |
| Small Area Mental Health Index (SAMHI) (17) | 1166 | 0.93 | 0.77 | -0.94 | 4.5 | 0.25 | 0.23 | 0.25 | 0.28 | 0.23 | 0.30 | 0.31 | 0.19 | 0.21 | 0.09 | 0.63 | -0.23 | 0.53 | 0.51 | 0.02 | -0.15 | 1 |  |
| Total Resident Population (log) (18) | 1166 | 7.38 | 0.19 | 6.93 | 8.50 | 0.37 | 0.34 | 0.35 | 0.35 | 0.38 | 0.36 | 0.35 | 0.26 | 0.20 | 0.22 | 0 | 0.33 | 0.04 | 0.05 | 0.24 | 0.12 | -0.11 | 1 |

**Table S2:** Linear regression predicting calls for service within LSOAs

|  | **Model 1** | | | **Model 2** | | |
| --- | --- | --- | --- | --- | --- | --- |
|  | *Coefficients* | *CI* | *p* | *Coefficients* | *CI* | *p* |
| **Intercept** | -1.41 | -2.41 – -0.42 | **0.005** | -1.43 | -2.42 – -0.44 | **0.005** |
| Black Resident Population | 0.07 | 0.04 – 0.11 | **<0.001** | 0.08 | 0.04 – 0.11 | **<0.001** |
| Concentrated Deprivation | 0.18 | 0.14 – 0.22 | **<0.001** | 0.16 | 0.11 – 0.20 | **<0.001** |
| Residential Turnover | 0.09 | 0.04 – 0.13 | **<0.001** | 0.10 | 0.05 – 0.14 | **<0.001** |
| Single-headed households | -0.02 | -0.06 – 0.02 | 0.275 | -0.02 | -0.06 – 0.02 | 0.232 |
| Crime decile (2) | 0.24 | 1.44 – 1.53 | **<0.001** | 0.24 | 0.13 – 0.34 | **<0.001** |
| Crime decile (3) | 0.43 | 0.33 – 0.53 | **<0.001** | 0.42 | 0.31 – 0.52 | **<0.001** |
| Crime decile (4) | 0.52 | 0.42 – 0.62 | **<0.001** | 0.50 | 0.40 – 0.60 | **<0.001** |
| Crime decile (5) | 0.59 | 0.49 – 0.70 | **<0.001** | 0.57 | 0.46 – 0.68 | **<0.001** |
| Crime decile (6) | 0.73 | 0.63 – 0.84 | **<0.001** | 0.71 | 0.60 – 0.82 | **<0.001** |
| Crime decile (7) | 0.79 | 0.67 – 0.90 | **<0.001** | 0.76 | 0.64 – 0.87 | **<0.001** |
| Crime decile (8) | 0.92 | 0.81 – 1.04 | **<0.001** | 0.88 | 0.77 – 1.00 | **<0.001** |
| Crime decile (9) | 1.06 | 0.94 – 1.18 | **<0.001** | 1.01 | 0.89 – 1.14 | **<0.001** |
| Crime decile (10) | 1.13 | 1.00 – 1.26 | **<0.001** | 1.09 | 0.95 – 1.22 | **<0.001** |
| Aged 15-34 Resident Population | -0.00 | -0.05 – 0.05 | 0.997 | 0.00 | -0.05 – 0.06 | 0.913 |
| Male resident population | 0.02 | -0.01 – 0.05 | 0.175 | 0.02 | -0.01 – 0.05 | 0.183 |
| Population Density | -0.14 | -0.17 – -0.11 | **<0.001** | -0.14 | -0.17 – 0.11 | **<0.001** |
| Total Resident Population (log) | 1.03 | 0.89 – 1.16 | **<0.001** | 1.03 | 0.90 – 1.17 | **<0.001** |
| SAMHI index 2019 |  |  |  | 0.05 | 0.01 – 0.08 | **0.007** |
| Observations | 1166 | | | 1166 | | |
| R^2^ / R^2^ adjusted | 0.64 / 0.63 | | | 0.64 / 0.63 | | |

**Table S3:** Multinomial logistic regression predicting incident response (reference category: all other call grades)

|  | **Model 1** | | | | **Model 2** | | |
| --- | --- | --- | --- | --- | --- | --- | --- |
|  | *Odds Ratio* | *CI* | | *P* | *Odds Ratio* | *CI* | *P* |
| **Grade 1** |  |  |  | |  |  |  |
| **Intercept** | 0.08 | 0.07 – 0.11 | **<0.001** | | 0.08 | 0.06 – 0.10 | **<0.001** |
| Black Resident Population | 0.98 | 0.97 – 0.99 | **<0.001** | | 0.97 | 0.96 – 0.98 | **<0.001** |
| Concentrated Deprivation | 0.98 | 0.97 – 0.99 | **<0.001** | | 1.00 | 0.99 – 1.01 | 0.857 |
| Residential Turnover | 0.95 | 0.94 – 0.97 | **<0.001** | | 0.94 | 0.93 – 0.95 | **<0.001** |
| Single-headed households | 1.06 | 1.05 – 1.07 | **<0.001** | | 1.06 | 1.05 – 1.07 | **<0.001** |
| Crime decile (2) | 0.97 | 0.93 – 1.00 | 0.066 | | 0.97 | 0.93 – 1.01 | 0.119 |
| Crime decile (3) | 0.92 | 0.89 – 0.95 | **<0.001** | | 0.93 | 0.90 – 0.97 | **<0.001** |
| Crime decile (4) | 1.04 | 1.01 – 1.08 | **0.017** | | 1.06 | 1.02 – 1.09 | **0.001** |
| Crime decile (5) | 1.13 | 1.09 – 1.17 | **<0.001** | | 1.15 | 1.11 – 1.19 | **<0.001** |
| Crime decile (6) | 1.14 | 1.10 – 1.18 | **<0.001** | | 1.16 | 1.12 – 1.20 | **<0.001** |
| Crime decile (7) | 1.06 | 1.02 – 1.10 | **0.001** | | 1.09 | 1.05 – 1.12 | **<0.001** |
| Crime decile (8) | 1.12 | 1.08 – 1.15 | **<0.001** | | 1.16 | 1.12 – 1.20 | **<0.001** |
| Crime decile (9) | 1.14 | 1.11 – 1.18 | **<0.001** | | 1.19 | 1.15 – 1.23 | **<0.001** |
| Crime decile (10) | 1.19 | 1.15 – 1.24 | **<0.001** | | 1.24 | 1.20 – 1.29 | **<0.001** |
| Aged 15-34 Resident Population | 1.06 | 1.04 – 1.08 | **<0.001** | | 1.06 | 1.04 – 1.07 | **<0.001** |
| Male resident population | 1.02 | 1.02 – 1.03 | **<0.001** | | 1.02 | 1.02 – 1.03 | **<0.001** |
| Population Density | 0.96 | 0.95 – 0.97 | **<0.001** | | 0.96 | 0.95 – 0.97 | **<0.001** |
| Total Resident Population (log) | 1.13 | 1.09 – 1.16 | **<0.001** | | 1.13 | 1.09 – 1.16 | **<0.001** |
| SAMHI index 2019 |  |  |  | | 0.96 | 0.95 – 0.96 | **<0.001** |
| **Grade 2** |  |  |  | |  |  |  |
| **Intercept** | 1.05 | 0.89 – 1.22 | 0.580 | | 1.05 | 0.90 – 1.24 | 0.511 |
| Black Resident Population | 1.01 | 1.00 – 1.02 | **0.006** | | 1.01 | 1.00 – 1.02 | **0.001** |
| Concentrated Deprivation | 0.99 | 0.98 – 1.00 | **0.002** | | 0.98 | 0.98 – 0.99 | **<0.001** |
| Residential Turnover | 1.01 | 1.00 – 1.02 | 0.119 | | 1.01 | 1.00 – 1.02 | **0.030** |
| Single-headed households | 1.06 | 1.05 – 1.07 | **<0.001** | | 1.06 | 1.05 – 1.07 | **<0.001** |
| Crime decile (2) | 1.09 | 1.06 – 1.11 | **<0.001** | | 1.09 | 1.06 – 1.11 | **<0.001** |
| Crime decile (3) | 0.95 | 0.93 – 0.98 | **<0.001** | | 0.95 | 0.93 – 0.97 | **<0.001** |
| Crime decile (4) | 1.04 | 1.02 – 1.07 | **<0.001** | | 1.04 | 1.02 – 1.06 | **0.001** |
| Crime decile (5) | 1.06 | 1.04 – 1.09 | **<0.001** | | 1.06 | 1.04 – 1.08 | **<0.001** |
| Crime decile (6) | 1.09 | 1.07 – 1.12 | **<0.001** | | 1.09 | 1.06 – 1.11 | **<0.001** |
| Crime decile (7) | 1.05 | 1.03 – 1.08 | **<0.001** | | 1.04 | 1.02 – 1.07 | **<0.001** |
| Crime decile (8) | 1.07 | 1.05 – 1.10 | **<0.001** | | 1.06 | 1.04 – 1.09 | **<0.001** |
| Crime decile (9) | 1.09 | 1.07 – 1.12 | **<0.001** | | 1.08 | 1.06 – 1.11 | **<0.001** |
| Crime decile (10) | 1.15 | 1.13 – 1.18 | **<0.001** | | 1.14 | 1.11 – 1.17 | **<0.001** |
| Aged 15-34 Resident Population | 1.04 | 1.03 – 1.05 | **<0.001** | | 1.04 | 1.03 – 1.05 | **<0.001** |
| Male resident population | 1.02 | 1.02 – 1.03 | **<0.001** | | 1.02 | 1.02 – 1.03 | **<0.001** |
| Population Density | 0.98 | 0.97 – 0.98 | **<0.001** | | 0.98 | 0.97 – 0.98 | **<0.001** |
| Total Resident Population (log) | 0.93 | 0.91 – 0.95 | **<0.001** | | 0.93 | 0.91 – 0.95 | **<0.001** |
| SAMHI index 2019 |  |  |  | | 1.01 | 1.00 – 1.02 | **<0.001** |
| Observations | 1310574 | | | | 1310574 | | |
| R^2^ / R^2^ adjusted | 0.001 / 0.001 | | | | 0.001 / 0.001 | | |

**Table S4:** Binary logistic regression predicting TASER officer dispatch following a call for service

|  | **Model 1** | | | **Model 2** | | | **Model 3** | | | **Model 4** | | | **Model 5** | | |
| --- | --- | --- | --- | --- | --- | --- | --- | --- | --- | --- | --- | --- | --- | --- | --- |
|  | *Odds Ratios* | *CI* | *p* | *Odds Ratios* | *CI* | *p* | *Odds Ratios* | *CI* | *p* | *Odds Ratios* | *CI* | *p* | *Odds Ratios* | *CI* | *p* |
| **Intercept** | 0.40 | 0.33 – 0.48 | **<0.001** | 0.40 | 0.33 – 0.48 | **<0.001** | 0.27 | 0.22 – 0.34 | **<0.001** | 0.28 | 0.22 – 0.34 | **<0.001** | 0.29 | 0.23 – 0.36 | **<0.001** |
| Black  Resident Population | 0.95 | 0.95 – 0.96 | **<0.001** | 0.96 | 0.95 – 0.96 | **<0.001** | 0.95 | 0.94 – 0.96 | **<0.001** | 0.95 | 0.94 – 0.96 | **<0.001** | 0.95 | 0.94 – 0.95 | **<0.001** |
| Concentrated Deprivation | 1.03 | 1.02 – 1.03 | **<0.001** | 1.02 | 1.01 – 1.03 | **<0.001** | 1.04 | 1.03 – 1.05 | **<0.001** | 1.03 | 1.02 – 1.04 | **<0.001** | 1.05 | 1.04 – 1.06 | **<0.001** |
| Residential Instability | 1.00 | 0.99 – 1.01 | 0.570 | 1.01 | 1.00 – 1.02 | 0.300 | 1.03 | 1.02 – 1.04 | **<0.001** | 1.04 | 1.02 – 1.05 | **<0.001** | 1.03 | 1.01 – 1.04 | **<0.001** |
| Single-Headed Households | 1.07 | 1.06 – 1.08 | **<0.001** | 1.07 | 1.06 – 1.08 | **<0.001** | 1.02 | 1.02 – 1.03 | **<0.001** | 1.02 | 1.02 – 1.03 | **<0.001** | 1.02 | 1.01 – 1.03 | **<0.001** |
| Crime Decile (2) | 1.07 | 1.04 – 1.10 | **<0.001** | 1.07 | 1.04 – 1.10 | **<0.001** | 1.08 | 1.05 – 1.12 | **<0.001** | 1.08 | 1.05 – 1.12 | **<0.001** | 1.07 | 1.04 – 1.11 | **<0.001** |
| Crime Decile (3) | 0.95 | 0.92 – 0.98 | **<0.001** | 0.95 | 0.92 – 0.97 | **<0.001** | 0.98 | 0.95 – 1.01 | 0.202 | 0.98 | 0.95 – 1.01 | 0.145 | 0.97 | 0.94 – 1.00 | **0.028** |
| Crime Decile (4) | 1.01 | 0.98 – 1.03 | 0.605 | 1.00 | 0.98 – 1.03 | 0.766 | 0.99 | 0.96 – 1.02 | 0.386 | 0.98 | 0.95 – 1.01 | 0.267 | 0.97 | 0.94 – 1.00 | **0.045** |
| Crime Decile (5) | 1.05 | 1.02 – 1.07 | **0.001** | 1.04 | 1.01 – 1.07 | **0.003** | 1.00 | 0.97 – 1.04 | 0.787 | 1.00 | 0.97 – 1.03 | 0.933 | 0.98 | 0.95 – 1.01 | 0.287 |
| Crime Decile (6) | 1.02 | 0.99 – 1.05 | 0.161 | 1.01 | 0.99 – 1.04 | 0.275 | 0.97 | 0.94 – 1.00 | **0.023** | 0.96 | 0.93 – 0.99 | **0.010** | 0.94 | 0.92 – 0.97 | **<0.001** |
| Crime Decile (7) | 1.03 | 1.01 – 1.06 | **0.011** | 1.03 | 1.00 – 1.06 | **0.033** | 1.01 | 0.98 – 1.05 | 0.343 | 1.01 | 0.98 – 1.04 | 0.621 | 0.99 | 0.96 – 1.02 | 0.473 |
| Crime Decile (8) | 0.99 | 0.97 – 1.02 | 0.698 | 0.99 | 0.96 – 1.01 | 0.323 | 0.94 | 0.91 – 0.97 | **<0.001** | 0.93 | 0.90 – 0.96 | **<0.001** | 0.91 | 0.88 – 0.94 | **<0.001** |
| Crime Decile (9) | 1.00 | 0.98 – 1.03 | 0.841 | 0.99 | 0.97 – 1.02 | 0.690 | 0.94 | 0.91 – 0.97 | **<0.001** | 0.93 | 0.90 – 0.96 | **<0.001** | 0.91 | 0.88 – 0.94 | **<0.001** |
| Crime decile (10) | 0.99 | 0.97 – 1.02 | 0.556 | 0.98 | 0.96 – 1.01 | 0.258 | 0.90 | 0.87 – 0.93 | **<0.001** | 0.89 | 0.86 – 0.92 | **<0.001** | 0.88 | 0.86 – 0.91 | **<0.001** |
| Aged 15-34 Resident Population | 1.02 | 1.00 – 1.03 | **0.008** | 1.02 | 1.00 – 1.03 | **0.007** | 0.97 | 0.96 – 0.99 | **<0.001** | 0.97 | 0.96 – 0.99 | **<0.001** | 0.97 | 0.96 – 0.99 | **<0.001** |
| Male Resident Population | 1.02 | 1.01 – 1.02 | **<0.001** | 1.02 | 1.01 – 1.02 | **<0.001** | 1.01 | 1.00 – 1.01 | 0.106 | 1.01 | 1.00 – 1.01 | 0.110 | 1.01 | 1.00 – 1.01 | **0.032** |
| Population Density | 1.05 | 1.04 – 1.05 | **<0.001** | 1.05 | 1.04 – 1.05 | **<0.001** | 1.06 | 1.05 – 1.06 | **<0.001** | 1.06 | 1.05 – 1.06 | **<0.001** | 1.06 | 1.05 – 1.07 | **<0.001** |
| Total Resident Population (log) | 0.95 | 0.92 – 0.97 | **<0.001** | 0.95 | 0.92 – 0.97 | **<0.001** | 0.92 | 0.90 – 0.95 | **<0.001** | 0.92 | 0.90 – 0.95 | **<0.001** | 0.92 | 0.89 – 0.95 | **<0.001** |
| SAMHI index 2019 |  |  |  | 1.01 | 1.00 – 1.02 | **0.006** |  |  |  | 1.01 | 1.01 – 1.02 | **0.001** | 1.01 | 1.00 – 1.02 | **0.005** |
| GRADE1 Violent Crime |  |  |  |  |  |  | 2.27 | 2.26 – 2.28 | **<0.001** | 2.27 | 2.26 – 2.28 | **<0.001** | 2.27 | 2.25 – 2.28 | **<0.001** |
| GRADE1 Non-Violent |  |  |  |  |  |  | 2.66 | 2.64 – 2.67 | **<0.001** | 2.66 | 2.64 – 2.67 | **<0.001** | 2.66 | 2.64 – 2.67 | **<0.001** |
| GRADE2 Violent Crime |  |  |  |  |  |  | 2.43 | 2.42 – 2.44 | **<0.001** | 2.43 | 2.42 – 2.44 | **<0.001** | 2.43 | 2.42 – 2.44 | **<0.001** |
| GRADE2 Non-Violent |  |  |  |  |  |  | 3.08 | 3.06 – 3.10 | **<0.001** | 3.08 | 3.06 – 3.10 | **<0.001** | 3.08 | 3.06 – 3.10 | **<0.001** |
| GRADE3+4 Violent Crime |  |  |  |  |  |  | 1.27 | 1.26 – 1.27 | **<0.001** | 1.27 | 1.26 – 1.27 | **<0.001** | 1.27 | 1.26 – 1.27 | **<0.001** |
| Deprivation x SAMHI |  |  |  |  |  |  |  |  |  |  |  |  | 0.98 | 0.97 – 0.98 | **<0.001** |
| Observations | 1310574 | | | 1310574 | | | 1310574 | | | 1310574 | | | 1310574 | | |
| R^2^ Tjur | 0.002 | | | 0.002 | | | 0.257 | | | 0.257 | | | 0.257 | | |

**Table S5:** Multinomial logistic regression predicting UoF/TASER use following dispatch of TASER units (reference category: no UoF)

|  | **Model 1** | | **Model 2** | | | | **Model 3** | | | **Model 4** | | |
| --- | --- | --- | --- | --- | --- | --- | --- | --- | --- | --- | --- | --- |
|  | *Odds Ratio* | *CI* | *P* | *Odds Ratio* | *CI* | *P* | *Odds Ratio* | *CI* | *P* | *Odds Ratio* | *CI* | *P* |
| **UoF (ex. TASER)** |  |  |  |  |  |  |  |  |  |  |  |  |
| **Intercept** | 0.02 | 0.01 – 0.06 | **<0.001** | 0.02 | 0.01 – 0.06 | **<0.001** | 0.01 | 0.00 – 0.03 | **<0.001** | 0.01 | 0.00 – 0.03 | **<0.001** |
| Black Resident Population | 0.98 | 0.94 – 1.02 | 0.233 | 1.02 | 0.98 – 1.06 | 0.457 | 0.99 | 0.95 – 1.03 | 0.493 | 1.03 | 0.98 – 1.07 | 0.224 |
| Concentrated Deprivation | 0.97 | 0.93 – 1.02 | 0.242 | 0.88 | 0.84 – 0.93 | **<0.001** | 0.97 | 0.92 – 1.01 | 0.109 | 0.87 | 0.83 – 0.92 | **<0.001** |
| Residential Instability | 1.00 | 0.94 – 1.05 | 0.922 | 1.04 | 0.98 – 1.10 | 0.190 | 1.03 | 0.98 – 1.09 | 0.245 | 1.08 | 1.02 – 1.15 | **0.006** |
| Single-headed households | 1.01 | 0.97 – 1.05 | 0.562 | 1.00 | 0.96 – 1.04 | 0.944 | 0.98 | 0.94 – 1.02 | 0.364 | 0.97 | 0.93 – 1.01 | 0.196 |
| Crime decile (2) | 0.92 | 0.77 – 1.11 | 0.378 | 0.91 | 0.76 – 1.09 | 0.297 | 0.95 | 0.79 – 1.14 | 0.592 | 0.93 | 0.77 – 1.11 | 0.412 |
| Crime decile (3) | 0.84 | 0.70 – 1.00 | 0.051 | 0.81 | 0.68 – 0.97 | **0.020** | 0.84 | 0.70 – 1.00 | 0.051 | 0.80 | 0.67 – 0.95 | **0.014** |
| Crime decile (4) | 0.95 | 0.80 – 1.12 | 0.545 | 0.91 | 0.77 – 1.08 | 0.287 | 0.95 | 0.80 – 1.12 | 0.524 | 0.89 | 0.75 – 1.05 | 0.173 |
| Crime decile (5) | 1.03 | 0.88 – 1.22 | 0.698 | 0.96 | 0.82 – 1.14 | 0.668 | 1.03 | 0.87 – 1.22 | 0.701 | 0.94 | 0.80 – 1.11 | 0.484 |
| Crime decile (6) | 1.00 | 0.85 – 1.18 | 0.978 | 0.94 | 0.80 – 1.11 | 0.479 | 1.02 | 0.86 – 1.21 | 0.809 | 0.94 | 0.79 – 1.11 | 0.443 |
| Crime decile (7) | 0.96 | 0.81 – 1.13 | 0.605 | 0.87 | 0.74 – 1.03 | 0.118 | 0.96 | 0.81 – 1.14 | 0.645 | 0.86 | 0.72 – 1.02 | 0.079 |
| Crime decile (8) | 1.14 | 0.97 – 1.34 | 0.115 | 0.96 | 0.81 – 1.13 | 0.635 | 1.11 | 0.95 – 1.31 | 0.198 | 0.93 | 0.79 – 1.10 | 0.403 |
| Crime decile (9) | 1.04 | 0.89 – 1.23 | 0.604 | 0.89 | 0.76 – 1.06 | 0.191 | 1.04 | 0.88 – 1.22 | 0.667 | 0.87 | 0.74 – 1.03 | 0.116 |
| Crime decile (10) | 1.15 | 0.97 – 1.36 | 0.109 | 0.98 | 0.82 – 1.16 | 0.807 | 1.11 | 0.94 – 1.31 | 0.233 | 0.94 | 0.79 – 1.11 | 0.457 |
| Aged 15-34 Resident Population | 1.17 | 1.10 – 1.25 | **<0.001** | 1.19 | 1.12 – 1.27 | **<0.001** | 1.14 | 1.07 – 1.21 | **<0.001** | 1.15 | 1.08 – 1.23 | **<0.001** |
| Male resident population | 1.02 | 0.99 – 1.05 | 0.180 | 1.02 | 0.99 – 1.05 | 0.237 | 1.02 | 0.99 – 1.06 | 0.168 | 1.02 | 0.99 – 1.05 | 0.229 |
| Population Density | 1.02 | 0.99 – 1.05 | 0.171 | 1.05 | 1.01 – 1.08 | **0.005** | 0.98 | 0.95 – 1.01 | 0.218 | 1.00 | 0.97 – 1.03 | 0.907 |
| Total Resident Population (log) | 1.01 | 0.87 – 1.16 | 0.941 | 1.03 | 0.89 – 1.19 | 0.708 | 1.02 | 0.88 – 1.18 | 0.785 | 1.01 | 0.88 – 1.17 | 0.875 |
| SAMHI index 2019 |  |  |  | 1.20 | 1.16 – 1.25 | **<0.001** |  |  |  | 1.20 | 1.16 – 1.25 | **<0.001** |
| GRADE 1 Violent Crime |  |  |  |  |  |  | 1.51 | 1.46 – 1.55 | **<0.001** | 1.51 | 1.46 – 1.55 | **<0.001** |
| GRADE 1 Non-Violent |  |  |  |  |  |  | 1.40 | 1.34 – 1.46 | **<0.001** | 1.40 | 1.35 – 1.47 | **<0.001** |
| GRADE 2 Violent |  |  |  |  |  |  | 1.29 | 1.23 – 1.35 | **<0.001** | 1.29 | 1.23 – 1.34 | **<0.001** |
| GRADE 2 Non-Violent |  |  |  |  |  |  | 1.15 | 1.07 – 1.23 | **<0.001** | 1.15 | 1.07 – 1.23 | **<0.001** |
| GRADE 3+4 Violent Crime |  |  |  |  |  |  | 1.05 | 0.99 – 1.11 | 0.099 | 1.05 | 0.99 – 1.11 | 0.107 |
| **TASER** |  |  |  |  |  |  |  |  |  |  |  |  |
| **Intercept** | 0.00 | 0.00 – 0.06 | **<0.001** | 0.00 | 0.00 – 0.07 | **<0.001** | 0.00 | 0.00 – 0.02 | **<0.001** | 0.00 | 0.00 – 0.03 | **<0.001** |
| Black Resident Population | 1.08 | 0.97 – 1.21 | 0.146 | 1.12 | 1.00 – 1.26 | **0.042** | 1.09 | 0.98 – 1.22 | 0.101 | 1.13 | 1.01 – 1.27 | **0.028** |
| Concentrated Deprivation | 0.95 | 0.84 – 1.07 | 0.396 | 0.86 | 0.75 – 0.99 | **0.035** | 0.93 | 0.83 – 1.05 | 0.266 | 0.86 | 0.75 – 0.98 | **0.029** |
| Residential Instability | 0.89 | 0.75 – 1.06 | 0.184 | 0.92 | 0.77 – 1.09 | 0.340 | 0.92 | 0.77 – 1.09 | 0.330 | 0.96 | 0.80 – 1.14 | 0.610 |
| Single-headed households | 1.10 | 0.98 – 1.24 | 0.092 | 1.11 | 0.99 – 1.25 | 0.079 | 1.08 | 0.96 – 1.22 | 0.184 | 1.08 | 0.96 – 1.21 | 0.208 |
| Crime decile (2) | 1.14 | 0.68 – 1.92 | 0.620 | 1.16 | 0.69 – 1.96 | 0.570 | 1.21 | 0.72 – 2.04 | 0.479 | 1.20 | 0.71 – 2.02 | 0.499 |
| Crime decile (3) | 1.13 | 0.69 – 1.86 | 0.622 | 1.10 | 0.67 – 1.82 | 0.700 | 1.14 | 0.69 – 1.89 | 0.600 | 1.10 | 0.66 – 1.81 | 0.720 |
| Crime decile (4) | 0.67 | 0.40 – 1.13 | 0.133 | 0.64 | 0.38 – 1.08 | 0.096 | 0.68 | 0.40 – 1.15 | 0.148 | 0.64 | 0.38 – 1.08 | 0.097 |
| Crime decile (5) | 1.26 | 0.78 – 2.03 | 0.341 | 1.20 | 0.74 – 1.94 | 0.466 | 1.29 | 0.79 – 2.08 | 0.305 | 1.18 | 0.73 – 1.92 | 0.498 |
| Crime decile (6) | 1.09 | 0.67 – 1.77 | 0.729 | 1.04 | 0.63 – 1.69 | 0.888 | 1.12 | 0.69 – 1.82 | 0.654 | 1.05 | 0.64 – 1.71 | 0.848 |
| Crime decile (7) | 0.81 | 0.49 – 1.34 | 0.411 | 0.75 | 0.45 – 1.25 | 0.272 | 0.83 | 0.50 – 1.37 | 0.465 | 0.75 | 0.45 – 1.25 | 0.265 |
| Crime decile (8) | 1.17 | 0.73 – 1.88 | 0.507 | 1.04 | 0.64 – 1.70 | 0.860 | 1.18 | 0.73 – 1.90 | 0.496 | 1.02 | 0.62 – 1.66 | 0.947 |
| Crime decile (9) | 1.06 | 0.66 – 1.71 | 0.820 | 0.92 | 0.56 – 1.50 | 0.738 | 1.04 | 0.64 – 1.69 | 0.864 | 0.91 | 0.56 – 1.49 | 0.708 |
| Crime decile (10) | 1.00 | 0.61 – 1.64 | 0.993 | 0.89 | 0.54 – 1.48 | 0.658 | 0.99 | 0.60 – 1.62 | 0.954 | 0.86 | 0.51 – 1.42 | 0.548 |
| Aged 15-34 Resident Population | 1.13 | 0.94 – 1.37 | 0.199 | 1.15 | 0.95 – 1.39 | 0.161 | 1.10 | 0.91 – 1.33 | 0.328 | 1.11 | 0.92 – 1.35 | 0.276 |
| Male resident population | 1.03 | 0.94 – 1.13 | 0.538 | 1.03 | 0.94 – 1.13 | 0.524 | 1.03 | 0.94 – 1.13 | 0.482 | 1.03 | 0.94 – 1.13 | 0.536 |
| Population Density | 1.06 | 0.97 – 1.16 | 0.194 | 1.07 | 0.98 – 1.17 | 0.120 | 1.01 | 0.93 – 1.11 | 0.769 | 1.03 | 0.94 – 1.13 | 0.543 |
| Total Resident Population (log) | 0.99 | 0.66 – 1.50 | 0.980 | 0.99 | 0.66 – 1.50 | 0.965 | 1.03 | 0.68 – 1.55 | 0.900 | 1.01 | 0.67 – 1.53 | 0.959 |
| SAMHI index 2019 |  |  |  | 1.16 | 1.04 – 1.30 | **0.008** |  |  |  | 1.16 | 1.04 – 1.29 | **0.009** |
| GRADE 1 Violent Crime |  |  |  |  |  |  | 1.57 | 1.43 – 1.72 | **<0.001** | 1.57 | 1.43 – 1.72 | **<0.001** |
| GRADE 1 Non-Violent |  |  |  |  |  |  | 1.55 | 1.35 – 1.77 | **<0.001** | 1.55 | 1.36 – 1.77 | **<0.001** |
| GRADE 2 Violent Crime |  |  |  |  |  |  | 1.32 | 1.15 – 1.51 | **<0.001** | 1.32 | 1.15 – 1.51 | **<0.001** |
| RADE 2 Non-Violent |  |  |  |  |  |  | 1.12 | 0.89 – 1.41 | 0.316 | 1.12 | 0.89 – 1.41 | 0.321 |
| GRADE 3+4 Violent Crime |  |  |  |  |  |  | 0.99 | 0.81 – 1.22 | 0.926 | 0.99 | 0.80 – 1.22 | 0.916 |
| Observations | 275813 | | 275813 | | | | 275813 | | | 275813 | | |
| R^2^ / R^2^ adjusted | 0.005 / 0.005 | | 0.006 / 0.006 | | | | 0.057 / 0.057 | | | 0.058 / 0.058 | | |

***
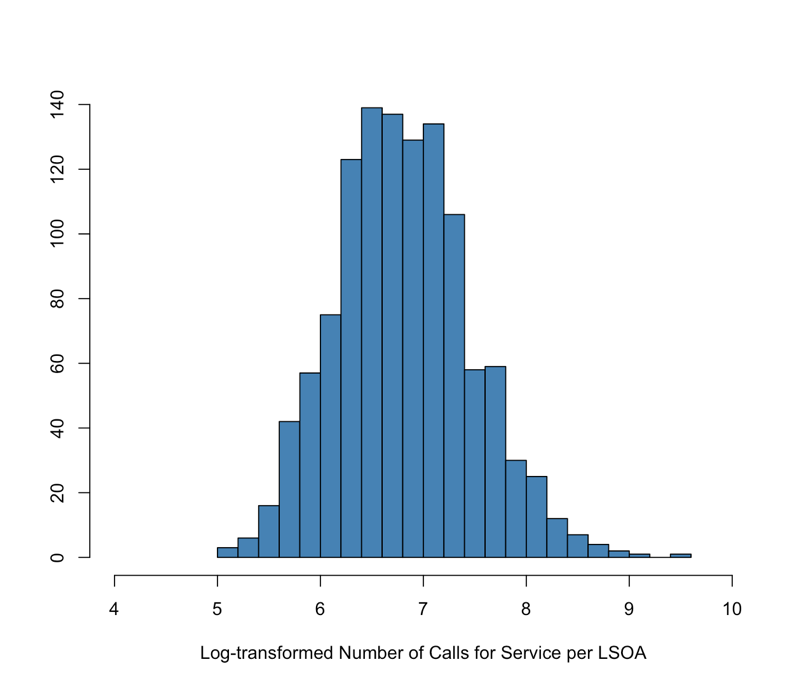
*Figure S1:** Log-transformed calls for service per LSOA

1. Three lockdown periods were examined and defined as follows: the first lockdown from 26/03/2020 to 23/06/2020, the second lockdown from 05/11/2020 to 02/12/2020, and the third lockdown from 06/01/2021 to 19/07/2021 (see: Institute for Government 2020). [↑](#footnote-ref-1)
2. Although incorporating lockdown periods into the model yields nearly identical results to the baseline, our analysis indicates that the first and second lockdowns are associated with small yet significant shifts in the odds of calls being graded 1 or 2 compared to other grades. As this is beyond the scope of this paper these findings are not discussed in the paper, but further details are available on request. [↑](#footnote-ref-2)
